# Mapping the cellular response to electron transport chain inhibitors reveals selective signaling networks triggered by mitochondrial perturbation

**DOI:** 10.1007/s00204-021-03160-7

**Published:** 2021-10-13

**Authors:** Wanda van der Stel, Huan Yang, Nanette G. Vrijenhoek, Johannes P. Schimming, Giulia Callegaro, Giada Carta, Salihanur Darici, Johannes Delp, Anna Forsby, Andrew White, Sylvia le Dévédec, Marcel Leist, Paul Jennings, Joost B. Beltman, Bob van de Water, Erik H. J. Danen

**Affiliations:** 1 Division of Drug Discovery and Safety, Leiden Academic Centre of Drug Research, Leiden University, Leiden, The Netherlands; 2 Division Molecular and Computational Toxicology, Vrije University Amsterdam, Amsterdam, The Netherlands; 3 Chair for In Vitro Toxicology and Biomedicine, Department Inaugurated by the Doerenkamp-Zbinden Foundation, University of Konstanz, Konstanz, Germany; 4 Department of Biochemistry and Biophysics, Stockholm University, Stockholm, Sweden; 5 Unilever, Bedfordshire, UK

**Keywords:** Mitochondrial toxicity, ETC complex inhibitors, High-content imaging, TempO-Seq, DILI

## Abstract

Mitochondrial perturbation is a key event in chemical-induced organ toxicities that is incompletely understood. Here, we studied how electron transport chain (ETC) complex I, II, or III (CI, CII and CIII) inhibitors affect mitochondrial functionality, stress response activation, and cell viability using a combination of high-content imaging and TempO-Seq in HepG2 hepatocyte cells. CI and CIII inhibitors perturbed mitochondrial membrane potential (MMP) and mitochondrial and cellular ATP levels in a concentration- and time-dependent fashion and, under conditions preventing a switch to glycolysis attenuated cell viability, whereas CII inhibitors had no effect. TempO-Seq analysis of changes in mRNA expression pointed to a shared cellular response to CI and CIII inhibition. First, to define specific ETC inhibition responses, a gene set responsive toward ETC inhibition (and not to genotoxic, oxidative, or endoplasmic reticulum stress) was identified using targeted TempO-Seq in HepG2. Silencing of one of these genes, NOS3, exacerbated the impact of CI and CIII inhibitors on cell viability, indicating its functional implication in cellular responses to mitochondrial stress. Then by monitoring dynamic responses to ETC inhibition using a HepG2 GFP reporter panel for different classes of stress response pathways and applying pathway and gene network analysis to TempO-Seq data, we looked for downstream cellular events of ETC inhibition and identified the amino acid response (AAR) as being triggered in HepG2 by ETC inhibition. Through *in silico* approaches we provide evidence indicating that a similar AAR is associated with exposure to mitochondrial toxicants in primary human hepatocytes. Altogether, we (i) unravel quantitative, time- and concentration-resolved cellular responses to mitochondrial perturbation, (ii) identify a gene set associated with adaptation to exposure to active ETC inhibitors, and (iii) show that ER stress and an AAR accompany ETC inhibition in HepG2 and primary hepatocytes.

**Supplementary Information:**

The online version contains supplementary material available at 10.1007/s00204-021-03160-7.

## Introduction

Accumulating evidence indicates that perturbation of mitochondria plays a role in the development of organ toxicity (Will and Dykens 2014, Dykens et al. 2007, Dykens and Will 2007). Disturbance of mitochondria upon chemical exposure has been monitored in the past based on changes in major functions of the mitochondria, including mitochondrial respiration and mitochondrial membrane potential (MMP) in both intact cells and isolated mitochondria (Porceddu et al. 2012, Rana et al. 2019, Zhang et al. 2009). The assay outcomes are predictive for the occurrence and potency of the interaction between chemical and target. Nevertheless, assessment of the mitochondrial status alone does not cover all toxicity-associated abnormalities sufficiently. Unraveling the interaction between mitochondrial perturbation and cellular responses can shed light on the eventual chemical-induced adversity at organ level.

Mitochondria are the organelles responsible for energy metabolism through oxidative phosphorylation (OXPHOS), hormone synthesis and metabolism. Bidirectional signaling between mitochondria and the nucleus enables rapid feedback concerning the metabolic and energetic needs of both compartments (Han et al. 2013, Monaghan and Whitmarsh 2015, Chandel 2014, Barbour and Turner 2014, Da Cunha et al. 2015). During oxidative phosphorylation, electrons flow through an electron transport chain (ETC) involving a series of enzyme complexes located in the mitochondrial double-layered membrane, and ultimately, release the energy stored in fats and carbohydrates to produce ATP. This process relies on an MMP (i.e., a proton gradient leading to concentration and charge imbalance across the mitochondrial membrane), which is generated by proton pumps including ETC complexes I, III and IV (CI, CIII and CIV). Complex II (CII) represents an alternative entry point into the ETC in addition to CI, but is itself not a proton pump. CIV transfers the electrons to oxygen (and pumps protons), and complex V (CV) is the enzyme that uses the energy that can be extracted from the MMP to convert ADP into ATP (Alberts et al. 2002).

Chemicals can perturb mitochondrial functioning via direct interaction with mitochondrial targets, or indirect via deprivation of building blocks and nutrients (Heiden et al. 2009). In general, cells possess an arsenal of adaptive stress response mechanisms to cope with toxic insults that give rise to among others reactive oxygen species (Sies et al. 2017), cytoplasmic unfolded proteins (Ron and Walter 2007), and DNA damage (Giglia-Mari et al. 2011). In addition, cells have mitochondrial damage-specific responses including upregulation of mitochondrial biogenesis (Jornayvaz and Shulman 2010, Hock and Krali 2009), induction of mitochondrial-specific unfolded protein response (UPR) (Münch 2018, Qureshi et al. 2017), adaptation of mitochondrial fission and fusion (Westermann 2010, Youle and Bilek 2012), and removal of damaged mitochondria by mitophagy (Youle and Narendra 2011, Hamacher-Brady and Brady 2016). The outcome of chemical-induced mitochondrial perturbation depends on the ability of cells to adapt and switch to alternative energy production via glycolysis (Merry and Ristow 2016). In the case that the cell cannot recover from the mitochondrial insult, apoptosis and/or necrosis will be induced to eliminate the damaged cells (Bock and Tait 2020). Large numbers of apoptotic or necrotic cells will result in tissue damage and ultimately organ failure as seen for instance in drug-induced liver injury (DILI) (Pessayre et al. 2012).

Combining the assessment of mitochondrial functionality with markers for a variety of cellular end points will generate information feeding into a mechanistic assessment of mitochondrial-related organ toxicity (van der Stel et al. 2020). Especially, time- and concentration-resolved exposure data can link the various involved processes, providing in-depth mechanistic understanding and distinguish lethal from adaptive cellular responses to chemical exposures.

In this study, we systematically assessed the changes in mitochondrial and cellular signaling upon exposure to a panel of ETC inhibitors using HepG2 cells. We unravel quantitative, time- and concentration-resolved mitochondrial and cellular responses to ETC inhibition providing mechanistic insight into mitochondrial toxicity. Using TempO-Seq, we first studied ETC inhibition-specific responses and identify a gene set that is induced selectively upon exposure to ETC CI and CIII inhibitors, which could be used to flag compounds for mitochondria-related toxicity. Assessment of downstream cellular events of ETC inhibition using a HepG2 GFP reporter panel for different classes of stress response pathways and applying pathway and gene network analysis to TempO-Seq data, we identify the amino acid response (AAR) as triggered in HepG2 by ETC inhibition. Through *in silico* approaches we provide evidence indicating that a similar AAR is associated with exposure to mitochondrial toxicants in primary hepatocytes (PHHs).

## Materials and methods

### Chemicals

All tested chemicals were purchased via the European Union Reference Laboratory for alternatives to animal testing (Joint Research Centre, Ispra, Italy) and stored as stock solutions between 10 and 100 mM in dimethyl sulfoxide (DMSO) at − 80 °C until use. Treatment solutions were created in appropriate medium (DMSO 0.1% (v/v)) on the day of exposure. The selected chemicals included complex I inhibitors capsaicin (Cat. No. M2028), deguelin (D0817), fenazaquin (31635), fenpyroximate (31684), pyridaben (46047), pyrimidifen (35999), rotenone (R8875), tebufenpyrad (46438); complex II inhibitors carboxin (45371), fenfuram (45486), flutolanil (N12004), mepronil (33361), thifluzamide (49792) and complex III inhibitors antimycin A (A8674), azoxystrobin (3167), cyazofamid (33874), fenamidone (33965), kresoxim-methyl (37899), picoxystrobin (33568), pyraclostrobin (33696), trifloxystrobin (46477). The included stress model compounds were CDDO-me (Cayman chemical; 11883), cisplatin (Ebewe pharma; 95199306), TNFα (R&D System-BioTechne; 210-TA-100) and tunicamycin (Merck; T7765). Seahorse experiments in HepG2 and RPTEC cells for these compounds have been previously published (van der Stel et al. 2020).

### Cell culture

HepG2 cells (ATCC; American Type Culture Collection, Wesel, Germany) were cultured in Dulbecco’s modified Eagle’s medium (DMEM) (Fisher Scientific, 11504496), supplemented with 10% (v/v) fetal bovine serum, 25 U/ml penicillin and 25 μg/mL streptomycin (FBS; South American, Fisher Scientific, S181L-500 & PenStrep, Fisher Scientific, 15070–063). Maintenance conditions were 37 °C in a 5% CO_2_ humidified atmosphere.

For experiments under conditions preventing glycolysis, medium was either replaced 1 day before the chemical exposures by glucose-free DMEM (Fisher Scientific, 11966–025) supplemented with 10 mM galactose (Sigma, G5388-100G) and 1 mM sodium pyruvate (Sigma, P2256-100g), or was enriched by medium containing 10 mM 2-deoxyglucose (Sigma-Aldrich, D8375-5G) at the moment of exposure.

### Generation of ATP biosensor cells

ATP dynamics was monitored using HepG2 cells expressing ATP biosensors located in the mitochondria (Ateam1.03) or cytoplasm (mitAT1.03). For this, cDNA constructs were provided by Hiromi Imamura (Precursory Research for Embryonic Science, Japan Science and Technology Agency) (Imamura et al. 2009) (Suppl. table 1). Constructs were introduced into HepG2 cells using lipofectamine2000 (INT) (Fisher Scientific, 11668–027). 8 µg DNA was combined with 10 µL of lipofectamine2000 in 500 µL serum free medium (SFM) to transfect 2*10^6 cells in a 6 cm dish. Cells were placed on G418 selection (PAA/Brunschwig chemie, P31-011) at 0.25 mg/ml and upon reaching confluency transferred to 10 cm dishes. Cells were kept on further selection at 0.5 mg/ml until colonies started to form. Colonies were picked, expanded and frozen to create a batch for usages (Suppl. Figure 1A, B and C). Cells stably expressing H2B-RFP and either a CFP or a YFP construct were kindly provided by Dr. Y. Zhang, LACDR, Leiden University, NL and used to adjust imaging settings (Suppl. Figure 1D). Localization of the FRET probes to their respective subcellular compartment (cytoplasm or mitochondria) was validated by microscopy.

### Confocal live cell imaging

*Mitochondrial membrane potential*: MMP was assessed using Rhodamine123 (Rho123, Sigma-Aldrich, R8004) by live confocal imaging. HepG2 cells were seeded in 384-wells μCLEAR^®^ black plate (Greiner Bio-One, 781 091) at a density of 10,000 cells/well. Two days after seeding, cells were stained with 200 ng/μL Hoechst 33342 (Life technologies, H1399) and 1 μM Rho123. After 60–75 min incubation at 37 °C the medium was refreshed into complete DMEM containing 0.2 μM Rho123, 100 nM propidium iodide (PI) (Sigma-Aldrich, P4170) and the desired concentration of the test chemicals. The signal intensity of Hoechst, Rho123 and PI (excitation wavelength respectively 408, 488 and 561 nm) were monitored hourly for 24 h. Note: the 24 h time point MMP data, but not the full-time kinetics, have been previously published (van der Stel 2020). Kinetic MMP data also serve as input for the development of a dynamic mathematical model; Yang et al., manuscript submitted.

*GFP-BAC reporters*: cellular stress response activation was evaluated using HepG2 BAC-GFP reporter cell lines (ATF4-GFP, BIP-GFP, CHOP-GFP, P21-GFP, SRXN1-GFP and XBP1-GFP) (Wink et al. 2017). Cells were plated in 384-wells μCLEAR^®^ black plate at a density of 10,000 cells/well. One day after seeding, cells were O/N stained with Hoechst 33342 and the medium was subsequently refreshed into complete DMEM containing the desired concentration of the test chemical. The signal intensity of Hoechst and GFP (408 and 488 nm) were monitored at 24, 48 and 72 h.

*Confocal live cell imaging of ATP biosensor*: cells stably transfected with cytoplasmic or mitochondrial ATP biosensors were seeded in 96-well μCLEAR^®^ black (Greiner Bio-One, 655090) at a density of 20,000 cells/well. Two days post-seeding the cells were exposed to a concentration range of the test chemicals. The exposures were performed using DMEM without phenol red to improve signal-to-noise ratio (DMEM (Thermos Fisher, 12196590), supplemented with 10% (v/v) FBS, 25 U/mL penicillin, 25 mg/mL streptomycin, 1 mM sodium pyruvate (Fisher Scientific, 11360070) and 4 mM l-glutamine (Fisher Scientific, 25030081). The signal intensity was monitored live every 5 min starting with untreated condition and followed by 2 h exposure with the desired test chemical. The ATP biosensors were excited at 408 nm and the FRET ratio was determined based on the emission at 408 and 488 nm.

All imaging was performed using a 20 × objective on a Nikon TiE2000 with perfect Focus System, automated-stage, and controlled temp/CO_2_ incubator (Nikon, Amsterdam, The Netherlands).

### Image analysis for MMP and BAC-reporter data

Object identification and signal quantification were performed using CellProfiler version 2.1.1 (Kamentsky et al. 2011). A segmentation module (Di et al. 2012) was used to segment nuclei objects based on the Hoechst signal. The cytoplasmic area was defined as the area around the nucleus to a maximal distance of 10 pixels (12.3 μM) or half the distance to the border of a neighboring cell’s nucleus. The signal intensity of Rho123, BIP-GFP, SRXN1-GFP and XBP1-GFP was quantified as the integrated pixel intensity in the cytoplasmic area. The signal intensity of ATF4-GFP, CHOP-GFP and P21-GFP was quantified as the integrated pixel intensity in the nuclear area. Nuclei were considered PI positive when the overlap of the nucleus with a PI object is larger than 10% of the nucleus area. All CellProfiler results were stored in HDF5 files and subsequently the data was extracted for further processing and visualization using in-house-developed R scripts (run in Rstudio (Boston, USA) (Rstudio Team 2016)) and the following packages: rhdf5, data.table, plyr, dplyr, tydr, ggplot2, reshape2, stringr, shiny, ggvis, gridExtra and doParallel (Dowle and Srinivasan 2021; Wickham 2007, 2011, 2016, 2019, 2020; Wickham et al. 2021; Chang and Wickham 2020; Chang et al. 2021; Auguie 2017, Weston and Microsoft corporation 2020).

### Image analysis for ATP biosensor data

The intensity quantification of the 408 and 488 nm emission images was performed using Ilastik version 1.1.9 (Berg et al. 2019, Sommer et al. 2011) and CellProfiler version 2.1.1. Background and foreground labels were based on manual curation of representative images of the 488 nm images and used for the creation of binary images of all conditions using Ilastik (Suppl. Figure 1E). The 408 and 488 nm intensity was monitored using CellProfiler in the region defined by the binary mask created with Ilastik. All CellProfiler results were saved as Excel file and further processed in R.

### ATPlite assay and analysis

ATP levels were assessed in whole cell lysates or in mitochondria after 2 h and 24 h exposures to chemicals. HepG2 cells were seeded in 96-wells μCLEAR^®^ black plate at a density of 20,000 cells/well. The cells were stained with Hoechst33342 for 60 min, followed by exposure. 1 h before the end of the exposure period the complete well was imaged with a 10 × objective and 7 × 6 montage using epifluorescence on a Nikon TiE2000 microscope with perfect focus system and xy-stage. After elapse of the exposure period, ATPlite 1-step Luminescence Assay reagent (PerkinElmer, 6016731) was added (1:1), followed by 2 min shaking and subsequent luminescence assessment using a FluoStar Optima plate reader (BMG Labtech). The epi-fluorescent pictures were used to normalize the data to the number of cells for each condition. Nuclear counting was performed using an in-house created macro for ImagePro software version 7.01 (Media Cybernetics). The macro performed watershed-based intensity segmentation after background correction (flatten function and edgefilter). The segmented objects were filtered for size and shape using the following parameters: Edgefilter = 3, RemoveNarrowObjects = TRUE, Min-area = 15 pixels, Max-area = 4000 pixels, Intensity threshold = 1000 and Mean-Intensity = 0.1.

### ATP levels in mitochondria

Mitochondrial-specific ATP was assessed after permeabilizing the cell membranes (Zoeteweij 1994) (supplementary Fig. 2F). After the desired exposure period Hanks’ buffer (Thermo Fisher 14175053) was supplemented with 5 mM HEPES, 250 mM sucrose (Thermo fisher s8600/63), 25 mM TRIS, 3 mM EGTA (Sigma-Aldrich 4378), 5 mM MgCl_2_ (Sigma-Aldrich 8266), 5 mM succinate (Sigma-Aldrich S2378) and 5 mM glutamate (Sigma-Aldrich G5889) (37 °C, pH 7.3). 150 µM digitonin (Sigma-Aldrich D5628) was added to permeabilize the cell membranes. After 30–45 s exposure, the buffer was replaced by PBS and the ATPlite 1step Luminescence Assay was performed as described above. The membrane permeabilization protocol was validated using confocal imaging. For this, cells were co-stained with Hoechst and 0.5 µM Rho123 and/or 0.05 µM Calcein–AM (VWR, 734–1434), to assess mitochondrial integrity (digitonin exposure should not affect the Rho123 intensity; Suppl. Figure 1F and G) and loss of cell membrane integrity (digitonin exposure should result in the loss of Calcein-AM signal; Suppl. Figure 1F, H and I). The Hoechst (408 nm) and Rho123/Calcein-AM (488 nm) signal intensity was monitored live every 10 s just before and after addition of digitonin using a 20 × objective on a Nikon TiE2000 with perfect focus system, automated-stage, and controlled temp/CO_2_ incubator (Nikon, Amsterdam, The Netherlands).

### Transcriptomics

HepG2 cells were plated into 96 wells plates (Costar, 3599) at a density of 50,000 cells/well. One day post-seeding medium was refreshed with complete DMEM containing the test chemicals. Plates were sealed with a gas-permeable seal (IST, IST-124-080SS). After 24 h exposure, the wells were washed once with ice-cold PBS (Sigma, D8537-500 ml), lysed using TempO-Seq lysis buffer (Bioclavis) for 15 min at RT, and subsequently stored at – 80 °C until shipment to BioClavis for TempO-Seq analysis (Yeakley et al. 2017, Limonciel et al. 2018). TempO-Seq was performed using a panel of probes targeting the “S1500 +  + ” gene list established by the EU-ToxRisk consortium (https://www.eu-toxrisk.eu), which covers the S1500 + sentinel gene list from the U.S. Tox21 Federal collaboration (Mav et al. 2018) and 587 additional probes including genes known to be affected in response to toxic insults and tissue relevant markers (Suppl. Table 2).

Expression data were returned by BioClavis as counts per probe per treatment. An in-house R script was developed to perform count normalization and determine differential gene expression. The script includes the following steps: (1) load data and metadata, (2) determine library size (total number of reads per sample) and remove samples with a library size below 100.000 reads, (3) use the DESeq2 function to normalize counts (per probe calculate a ratio = *raw counts/geometric mean of that probe*, after which the median of the raw counts of each probe is divided by the median of all probe ratios for a treatment, and calculate differentially expressed genes (DEGs) considering cell line, treatment, concentration and time point), (4) create matrix of all comparisons between vehicle control and any of the other treatments. The analysis utilized the following packages: gridExtra, stringr, ggplot2, pheatmap, reshape2, RColorBrewer, plyr, dplyr, tidyr, colorspace, scales, data.table, DESeq2, compare, readxl, PoiClaClu, hexbin, ggalt, vsn, org.GS.eg.db, annotationDbi (Kolde 2019; Neuwirth 2014; Zeileis et al. 2020; Wickham and Bryan 2019; Wickham 2019, 2020; Wickham and Seidel 2020; Love et al. 2014; Murell et al. 2015; Witten 2019, Carr 2021, Rudis et al. 2017, Huber et al. 2002, Carlson 2019; Pagès et al. 2020).

### BMD express analysis: Williams trend test and BMD calculations

Input for the BMD express software (version 2.3) (Phillips et al. 2019) was the log2 of the normalized data. A Williams trend test was used to determine concentration responses (10,000 permutations; no filters). The output of the Williams trend test included a *p* value per probe per treatment. Subsequently bench mark dose (BMD) (Haber et al. 2018) values were determined based on the best model fit (lowest akaike information criterion (AIC)). The following parametric models were used to derive dose response curves: power, linear, polynomial (2 parametric), Hill, and exponential (2–5 parametric) (maximum iterations = 250; confidence = 0.095; BMR factor = 1SD). The output of the BMD model fitting provided a “best model fit” per probe per treatment.

### Pathway analysis

Gene ontology (GO)-term enrichment analysis was performed using the GOrilla website (Eden et al. 2009). For the analysis, the background file consisted of Ensembl IDs of all unique genes in the EU-ToxRisk panel and the significant set consisted of genes with *p* adjusted < 0.05; log2FC threshold of < − 0.58 or > 0.58; Williams trend test *p* value < 0.05.

### Transcription factor (TF) enrichment analysis

A TF enrichment analysis was performed using the DoRothEA tool version 2 (Garcia-Alonso et al. 2018). The log2 normalized values were used as input for the analysis. For genes with multiple probes, an average fold change was calculated over all probes of the gene and used to determine *z*-scores (compared to DMSO). The Viper package was used to determine the TF enrichment including information with confidence set ABC (Alvarez et al. 2016). The Viper output consisted of a normalized enrichment score (NES) per transcription factor per treatment.

### Microarray data of HisOH in HepG2

Microarray results of HepG2 cell exposed to 5 mM HisOH for 4 h were used to assess possible similarities in the induction of signaling pathways between HisOH and ETC inhibitors (Shan 2010). The Affymetrix Human Genome U133 Plus 2.0 Array was used, and count files were stored at GEO (Number: GSE19495). We obtained log2FC and p-adjusted values using the GEO2R analysis provided by GEO. Values are log2FC of treated HepG2 versus medium control.

### TG-GATES data: gene expression analysis

Primary human hepatocyte (PHH) gene expression data was obtained from the open TG-GATES database: “Toxicogenomics Project and Toxicogenomics Informatics Project under CC Attribution-Share Alike 2.1 Japan” and processed as reported previously (Callegaro et al. 2021). Briefly, microarrays were jointly normalized using the Robust Multi-array Average (RMA) method (affy R package) (Gautier et al. 2004) and probes were mapped to gene IDs with BrainArray chip description file (CDF) version 20.^1^ Differential gene expression analysis was performed by building a linear model fit and computing the log-odds of differential expression by empirical Bayes moderation (limma R package) (Ritchie et al. 2015).

### Real-time PCR

HepG2 cells were seeded into 24-well plates (Costar) at a density of 200,000 cells/well. Two days post-seeding, the medium was changed to complete DMEM containing the desired test chemical. After 24 h exposure, the wells were washed with PBS and RNA was isolated using the NucleoSpin RNA kit (Marcherey-Nagel, 740955.25) according to the manufacturer’s protocol. cDNA was synthesized from 800 ng RNA per reaction using the RevertAid H Minus First Strand cDNA synthesis kit (Thermo Scientific, K1632). Real-time PCR was performed using SYBR Green (Applied Biosystems, A25742) and KiCqstart SYBR green primers (Sigma) (Suppl. Table 3) using the QuantStudio 6 Flex Real-Time PCR System (ThermoFisher Scientific).

### RNA interference

Transient knockdown of desired genes was achieved through reverse transfection with siGENOME Smartpool siRNAs (50 nM, from Dharmacon GE Healthcare). siRNAs were incubated with 0.3% INTERFERin transfection reagent (Westburg/PolyPlus, 409-50) for 20 min. Subsequently, cells were seeded on top at a density of 23,000 cells per well (in a 96 wells μCLEAR^®^ black plate). The medium was refreshed after 24 h. All follow-up assays were performed 72 h after transfection.

### Resazurin reduction assay

The cell viability assessment using the resazurin reduction readout was performed as previously described (Jennings et al. 2007, van der Stel et al. 2020). Briefly, after chemical exposure the medium from the cell culture was replaced with medium containing 44 µM resazurin. The conversion of resazurin to fluorescent resorufin was monitored after 1.5–2 h incubation at 37 °C in 5% CO_2_ humidified atmosphere in a plate reader at excitation/emission 540/590 nm.

### Phenomenological modeling

The phenomenological model for the MMP ($\Psi (t)$) data encompasses the following equation:

$$\Psi \left(t\right)=a\, exp \left(\frac{-t}{\tau }\right)+\left(1-a\right),$$

where $a$ denotes the maximal reduction of the MMP. The MMP starts at a value of $\Psi \left(0\right)=1$, and when $t \to \infty$, the MMP approaches $1-a$. We consider parameter $a$ to be chemical and concentration specific. The parameter τ denotes the MMP decay time constant, i.e., for large τ values the MMP drops slowly to the minimum (1-$a$). We consider τ to be only chemical specific, so its value is shared among the different concentrations.

We fitted the phenomenological model to the MMP data for each compound using the weighted least square approach, where *τ* and $a$ are free parameters to be tuned to minimize the cost function as a weighted sum of squared of difference between data and model predictions. To obtain a global optimum, we utilized 1000 sets of starting values for the parameters, which were sampled randomly as positive numbers. The resulting estimated $a$ values for all compounds were utilized during hierarchical clustering with the ward criterion (Ward 1963). Moreover, we studied the relation between the sum of the $a$ values for all concentrations of a compound and corresponding logP value for that compound. The logP values were obtained from the PubChem database using the python package pubchempy.^2^

### Statistical analysis

The R package DESeq2 was used to calculate the fold change compared to vehicle control per condition. The fold change values are represented with the standard error. *p* adjusted values per condition were also calculated using DESeq2 based on the Wald test and Benjamini Hochberg correction. The significance threshold for the gene expression data was set at *p* adjusted < 0.05.

A generalized linear model (glm) was used to compare the different variables (average abs log2F for yes/no in vivo mitochondrial toxicant + concentration (numeric) + time(numeric)) in the TG-GATES PHH dataset (package stats (R core team 2018)).

## Results

### Mitochondrial complex inhibitors differentially affect the MMP

**Fig. 1:**
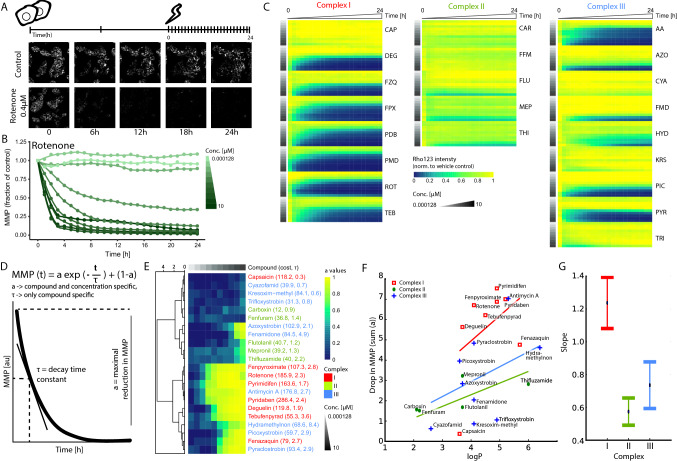
Effect of various agrochemical mitochondrial respiratory chain inhibitors on mitochondrial membrane potential dynamics. **A** Schematic representation of the experimental setup in HepG2 cells. Cells were seeded 2 days before exposure. At day 3, cells were stained with Hoechst33348 (nuclei) and Rho123 (MMP) followed by chemical exposure. Upon exposure, cells were monitored every hour for 24 h. Image panels demonstrate representative snapshots at five time points (0, 6, 12, 18 and 24 h) of Rho123 staining in vehicle control samples and upon exposure to 0.4 µM rotenone. **B** Quantification of one replicate showing Rho123 intensity over time upon exposure to a concentration range of rotenone as fraction of the vehicle control. **C** Heatmap including the Rho123 intensity over time upon exposure to a concentration range of 22 chemicals divided over CI (CAP = capsaicin, DEG = deguelin, FZQ = fenazaquin, FPX = fenpyroximate, PDB = pyridaben, PMD = pyrimidifen, ROT = rotenone, TEB = tebufenpyrad), CII (CAR = carboxin, FFM = fenfuram, FLU = flutolanil, MEP = mepronil, THI = thifluzamide) and CIII (AA = antimycin A, AZO = azoxystrobin, CYA = cyazofamid, FMD = fenamidone, HYD = hydra-methylnon, KRS = kresoxim-methyl, PIC = picoxystrobin, PYR = pyraclostrobin, TRI = trifloxystrobin) inhibitors. The values represent the geometric mean of four biological replicates, which is expressed as fraction of the measurements for the control condition. **D** Phenomenological model describing MMP dynamics measured using Rho123. The equation describes exponential decay toward a minimal MMP, with *τ* representing a chemical specific MMP decay time constant and *a* representing the maximal reduction of the MMP which is chemical and concentration dependent. **E** Clustering heatmap generated with the Wald algorithm showing the estimated ***a*** value per compound and per concentration for in total 22 complex inhibitors plus the cost and *τ* value per compound. **F** Correlation plot comparing the logP value with the sum of all determined *a* values per inhibitor. The logP values were collected from PubChem. The correlation line is an ordinary-linear-least square regression between logP and the sum of all *a* values of an inhibitor (*r*^2^ values: I = 0.899, II = 0.923 and III = 0.770). The Python package used for the regression line is statsmodels (Seabold and Perktold 2010). **G** Estimated regression line slopes (± standard error) for the data from F. Colored names (C, E), symbols (F) and error bars (G) denote (CI (red), CII (green) or CIII (blue) inhibitors)

To assess the effects of the various ETC inhibitors on the MMP dynamics, HepG2 cells were stained with Rho123 to monitor the MMP over a period of 24 h. The MMP decreased in a concentration- and time-dependent manner upon exposure to the CI inhibitor rotenone (Fig. 1A, B). A concentration range of various mitochondrial CI, CII and CIII inhibitors was evaluated for their effect on the MMP (Fig. 1C). Most complex I and III inhibitors, except for the weak CI inhibitor capsaicin (Delp et al. 2019, van der Stel et al. 2020), decreased the MMP in a concentration- and time-dependent manner. CII inhibitors, CIII inhibitors kresoxim-methyl and trifloxystrobin and the CIII inhibitor/uncoupler cyazofamid (van der Stel et al. 2020) only weakly affected the MMP at the highest concentration. Various highly potent CI inhibitors decreased the MMP already within the first 2 h of exposure.

To assess whether MMP dynamics were differentially perturbed by CI or CIII inhibitors, a phenomenological model was built and fitted to the data to capture compound- and concentration-dependent features of MMP decay (Fig. 1D and Suppl. Figure 2). In this way, we estimated the maximal reduction of the MMP (*a*) that depended on the chemical and its concentration. Hierarchical clustering using the estimated maximal reduction per compound and concentration resulted in two major clusters: potent inhibitors vs chemicals with low potency/no effect (Fig. 1E). Based on the maximal reduction it was not possible to distinguish CI inhibitors from CIII inhibitors. However, per group of inhibitors, the maximal MMP reduction clearly depended on the lipophilicity (logP) of the inhibitors, i.e., the drop in the MMP is highest for compounds with high logP values (Fig. 1F). In general, the MMP drop of CI inhibitors depended more strongly on the logP value than CII and CIII inhibitors (Fig. 1G). In summary, our measurements on MMP dynamics suggest that CI and CIII inhibitors are more potent than CII inhibitors, and that this is in part correlated with the high lipophilicity of specific compounds.

### Inhibition of glycolysis causes loss of viability in the presence of complex I and III, but not complex II inhibitors

**Fig. 2:**
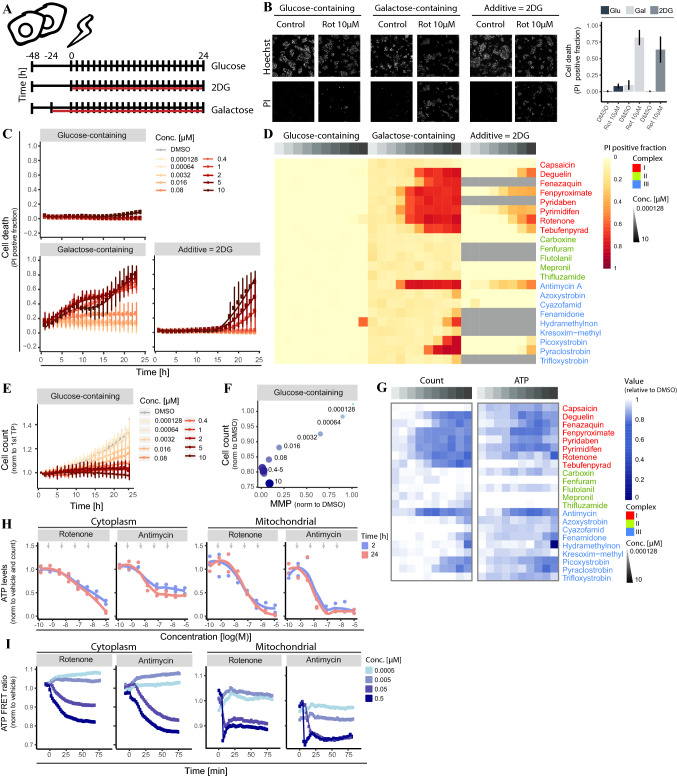
Effect of various agrochemical MRC inhibitors on cell viability and ATP levels. **A** Schematic representation of different culture conditions. For all conditions, cells were seeded in glucose-containing medium 2 days before the chemical exposure. (1) Glucose-containing medium: 2 days after seeding the medium is changed into new glucose-containing medium during the exposure. (2) Glucose-containing medium supplemented with 2DG: At the moment of chemical exposure 10 mM of 2DG was added into the wells. (3) Galactose-containing medium: Medium is refreshed into galactose-containing medium 1 day after seeding. 1 day later the chemical exposure is performed in galactose-containing medium. **B** Representative images (of Hoechst and PI) plus PI-positive fraction quantification of HepG2 cells exposed of 24 h to the vehicle control or 10 µM rotenone in 3 different medium conditions. **C** 24 h live cell death measurement based on PI positive fraction upon exposure to concentration range of rotenone in 3 medium conditions. Values are mean ± SD of 3 biological replicates. **D** Heatmap showing PI positive fraction upon exposure to concentration ranges of 22 complex inhibitors in three medium conditions (glucose-containing, galactose-containing and addition of 2DG). Values are a mean of three biological replicates. Gray cells in the 2DG medium condition were not tested. **E** Nuclear count data in glucose-containing medium upon exposure to a concentration range of rotenone. Values were normalized to the first time point and represent a mean ± SD of 3 biological replicates. **F)** Correlation plot comparing MMP at 24 h (geometric mean normalized to DMSO) to cell ratio between 1st and last time point (normalized to DMSO) after exposure to a concentration range of rotenone. Values are mean of four biological replicates. Labels represent corresponding concentration in µM. **G** Heatmap showing total cytoplasmic ATP levels and nuclear count ratio between first and last time point after exposure to concentration ranges of 22 complex inhibitors in glucose-containing medium. Values are normalized to vehicle control and represent a mean ± SD for two biological (ATP levels) or three biological (nuclear count) replicates. **H** Concentration–response curve of total ATP levels in the cytoplasm (left two panels) and in mitochondria (right two panels) comparing rotenone or antimycin exposure to vehicle control at 2 h and 24 h. Values are normalized to nuclear count and represent a biological triplicate ± SD Arrows depict the concentrations used in Fig. 2I. **I** Time response curve of ATP-FRET ratio in the cytoplasm (left two panels) and mitochondria (right two panels) comparing four indicated concentrations of rotenone or antimycin to vehicle control over a period of 2 h. Values are from 1 biological replicate, see supplementary Fig. 2D and E for second biological replicates

Like primary liver tissue, HepG2 cells can increase their anaerobic non-mitochondrial respiration rate using glycolysis and do this more extensively than primary liver cells (Rodriguez-Enriquez et al. 2001). We prevented the increase in glycolytic rate in response to mitochondrial insult by switching the cells from glucose- to galactose-containing medium or inhibited glycolysis using the competitive inhibitor 2-deoxyglucose (2DG) (Marroquin et al. 2007, Kamalian et al. 2015, Korga et al. 2019, Pietzke et al. 2014) (Fig. 2A). While HepG2 cells tolerated exposure to rotenone in glucose-containing medium, preventing the increase in glycolytic rates and inhibition of glycolysis itself sensitized these cells to adversity caused by CI inhibition as shown by propidium iodide staining (Fig. 2B). The use of galactose medium sensitized the cells to cell death to a larger extent and at earlier time points than the addition of 2DG to the medium (Fig. 2C). Perturbation of glycolytic capacity of the cell did lead to sensitization to cell death in response to both CI and CIII inhibitors, but no difference could be observed between the two inhibitor classes other than potency variability (Fig. 2D). No sensitization of HepG2 to CII inhibitors upon perturbation of the glycolytic capacity using either 2DG or galactose-containing medium was observed. These findings indicate that HepG2 cells can be stimulated to largely rely on mitochondrial respiration and under those conditions, inhibition of CI or CIII but not of CII reduces cell viability.

### Attenuation of mitochondrial ATP production and proliferation in presence of complex I and III, but not complex II inhibitors

The ability of HepG2 cells to tolerate ETC inhibition in glucose-containing medium allowed us to study the response to CI and CIII inhibitors in more detail at any desired moment after exposure. Despite the absence of cell death in glucose-containing medium (Fig. 2B), cell proliferation was attenuated in a concentration- and time-dependent manner by rotenone (Fig. 2E). The observed decrease in proliferation correlated to inhibition of MMP (Fig. 2F). Assessment of relative cell count upon exposure to the panel of CI, CII and CIII inhibitors also confirmed the correlation between a drop in proliferation and perturbation of the MMP (Fig. 2G left panel and Fig. 1C).

Next, the effect of CI and CIII inhibition on ATP concentration was evaluated in the cytoplasm and mitochondria using the ATPlite assay. Exposure to both CI (rotenone) and CIII (antimycin) inhibitors resulted in a concentration-dependent decrease of total cytoplasmic ATP levels at 2 h, which was still the case at 24 h. (Fig. 2H left two panels). The concentration-dependent decrease in ATP levels in the mitochondria resembled the pattern observed in the cytoplasm (Fig. 2H right two panels). We therefore only examined the total cytoplasmic ATP for the entire set of CI, CII and CIII inhibitors (Fig. 2G right panel). In general, CI and CIII inhibitor exposure resulted in a drop in ATP which correlated with decreased proliferation. CII inhibitors did not exhibit a pronounced concentration-dependent decrease.

Finally, to assess the dynamics of the ATP levels (sum of production and degradation) in the cytoplasm and in the mitochondria, cell lines were created by stable integration of ATP-FRET probes localized in the cytoplasm or mitochondria. These allowed dynamic monitoring of ATP changes upon chemical exposure as exemplified using CI (rotenone) and III inhibitors (antimycin) (Suppl. Figure 1A–D). Assessment of the temporal dynamics using both ATP-FRET probes over a period of 75 min confirmed the concentration-dependent decrease in ATP (Fig. 2I, Suppl. Figure 2D and E). Cytoplasmic ATP levels dropped between the moment of exposure and the following 75 min in a concentration-dependent manner for CI and CIII inhibitors rotenone and antimycin. The ATP drop in the mitochondrial compartment was much faster than in the cytoplasm and already reached a minimum within the first 10 min.

In summary, our measurements demonstrated that exposure to CI and CIII inhibitors in glucose-containing medium resulted in a quick concentration-dependent drop in cytoplasmic and mitochondrial ATP, which was correlated with the inhibition of MMP and, when glycolysis is suppressed, by inhibition of cell proliferation.

### A gene signature for mitochondrial toxicants

Having established that ETC CI and III inhibitors similarly attenuate MMP and mitochondrial ATP production, we collected targeted TempO-Seq transcriptomics data using the S1500 +  + probe set, which expands the U.S. Tox21 S1500 + sentinel list with probes targeting genes known to be affected in response to toxic insults and tissue-relevant markers to further analyze the cellular response toward ETC inhibition. A concentration-dependent increase in the number of differentially expressed genes (DEGs) was observed in response to CI and CIII inhibitors, whereas CII inhibitors did not affect overall gene expression (Suppl. Figure 3A). Notably, the potency difference between the various chemicals with respect to the number of DEGs correlated inversely with MMP perturbation (Suppl. Figure 3B). To evaluate if concentration-dependent regulation of individual genes was correlated to the effects of inhibitors on MMP, for each probe we determined the benchmark concentration (BMC = the lowest concentration at which the probe count was changed more than 1 × standard deviation (up or down) compared to the control condition). Indeed, accumulation plots of all BMC values per probe, measured upon exposure to the various ETC inhibitors, demonstrated an increase around the concentration at which also the MMP BMC was observed (Suppl. Figure 3C).

**Fig. 3:**
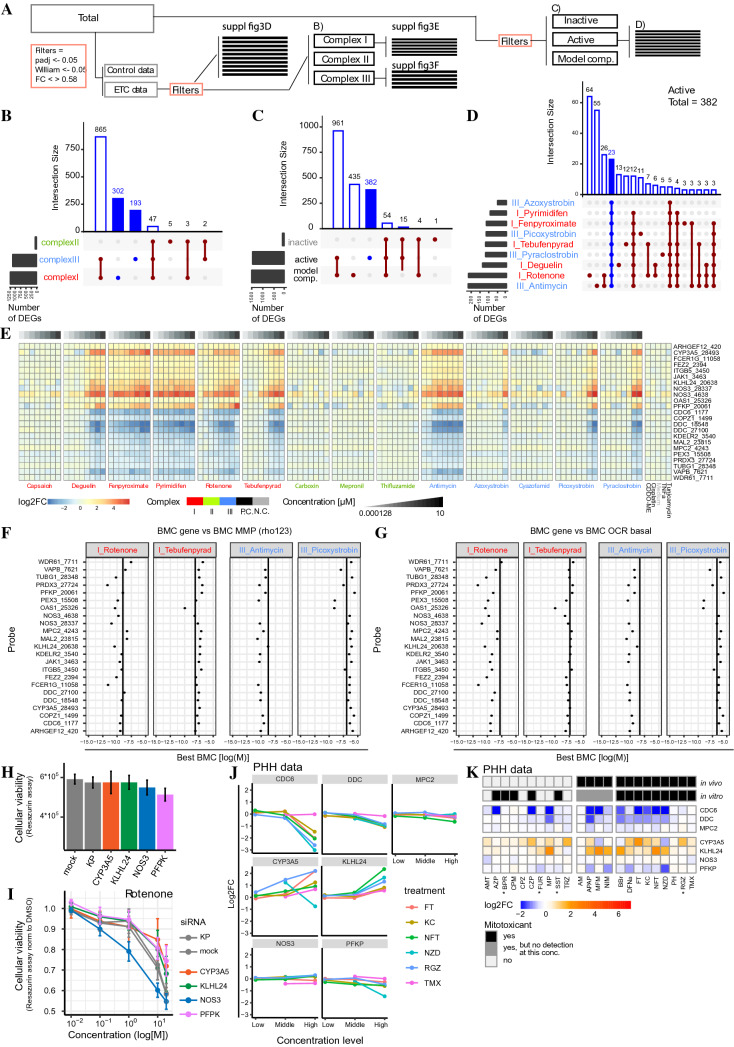
Predictive gene expression signature for MRC inhibition. **A** Schematic representation of the relationship between panels B–E. All plots only include genes with a padj < 0.05, log2FC > or < 0.58 and a *p* value < 0.05 in the Williams trend test. **B** Plot based on the gene expression data of all mitochondrial complex inhibitors and separating all genes per complex type. Horizontal bars at the left represent the total number of DEGs per treatment that meet the filtering criteria. Blue bars represent the group of genes unique for CI or CIII inhibition **C** Plot based on the gene expression data of all mitochondrial complex inhibitors plus all data from the four positive adaptive stress controls (60 nM CDDO-me, 1 mg/ml cisplatin, 10 ng/mL TNFα and 12 µM tunicamycin) and separating all genes active inhibitors, inactive inhibitors and positive adaptive stress controls). Horizontal bars at the left represent the total number of DEGs per treatment which meet the filtering criteria. Blue bar represents group of genes unique for all active complex inhibitors **D** Subset of 382 genes from panel D) separated per active mitochondrial inhibitor. The plot only represents groups of more than three genes. Horizontal bars at the left represent the number of DEGs per treatment which meet the filtering criteria and are in the set of 382 probes. Blue bar represents group of genes affected by all active inhibitors. **E** Heatmap with log2FC values upon exposure to a concentration range of 14 complex inhibitors and the positive adaptive stress controls (60 nM CDDO-me, 1 mg/ml cisplatin, 10 ng/mL TNFα and 12 µM tunicamycin) of the 23 genes affected by all active mitochondrial complex inhibitors and not the positive stress responses controls (from panel E). **F** Plot including per treatment (CI and CIII inhibitors) the BMD value of the Rho123 data (vertical line) plus the individual BMD value of the 23 probes at 24 h chemical exposure. Both BMD values are determined using BMDexpress software. **G** Plot including per treatment the BMD value of the OCR basal data ((van der Stel 2020), vertical line) plus the individual BMD value of the 23 probes. Both BMD values are determined using BMDexpress software. **H** Cellular viability monitored using resazurin reduction 72 h after siRNA transfection (CYP3A5, KLHL24, NOS3, PFKP or siRNA control: kinase pool (KP)) or negative control condition (mock). **I** Cellular viability monitored using resazurin reduction after 72 h siRNA transfection followed by a concentration range of rotenone for 24 h (CYP3A5, KLHL24, NOS3, PFKP or siRNA control: kinase pool (KP)) or negative control condition (mock). Values are normalized to treatment control (DMSO). **J** Log2FC data from the TG-GATES database for CDC6, DDC, MPC2, CYP3A5, KLHL24, NOS3 and PFPK in PHH upon 24 h exposure to three concentrations of DILI-inducing compounds know to also effect the ETC (Table 2). **K** Heatmap with log2FC values from the TG-GATES database for CDC6, DDC, MPC2, CYP3A5, KLHL24, NOS3 and PFPK upon 24 h exposure to the highest concentration available for DILI compounds classified by Eakins 2016 (Table 2). (* = exceptions are with middle concentration, because the highest concentration was more than 100-fold the cMax value). In vivo = classification based on literature search presented by Eakins 2016 and in vitro = classification based on results in HepG2 by Eakins 2016. Gray classification = concentration presented in the heatmap is lower than the mitotoxic concentration determined by Eakins in HepG2

We considered whether a signature of unique genes could achieve separation between the various mitochondrial complex inhibitors. To enable selection of candidate genes for the different complex inhibitors, DEGs were filtered based on p-adjusted values, fold change values, and their concentration–response correlation (William’s trend test) (Fig. 3A). Thereafter, a group of gene probes (299) was selected that was affected by any active ETC inhibitor (inactive = capsaicin, carboxin, cyazofamid, mepronil and thifluzamide) (Suppl. Figure 3D). We subdivided the probe set across CI, CII and CIII inhibitors and defined genes affected specifically by the CI or CIII inhibitors, respectively, 302 and 193 probes (Fig. 3B). Using this approach, no genes were identified that responded to all compounds within one class, but not to any compounds of another class (Suppl. Figure 3E and F).

While no subclass specific DEGs were found, the individual gene expression patterns could still be used to create a gene signature for mitochondrial perturbation. We subdivided the probe set along “active inhibitors” (CI: deguelin, fenpyroximate, pyrimidifen, rotenone, tebufenpyrad and CIII: antimycin, azoxystrobin, picoxystrobin, pyraclostrobin), “inactive inhibitors” with none to minimal effect upon proliferation (capsaicin, carboxin, cyazofamid, mepronil and thifluzamide) and “model compounds” (CDDO-me (oxidative stress response), cisplatin (DNA damage response), TNFα (inflammation) and tunicamycin (unfolded protein response)). This resulted in 382 probes affected specifically by one or more active mitochondrial toxicants and not by the positive controls or “inactive” inhibitors (Fig. 3C). 23 of these 382 probes were affected by all individual “active inhibitors” (Fig. 3D). The identified hits exhibited a concentration–response relationship upon exposure to the various “active inhibitors” (Fig. 3E) and a BMC value which was less than or equal to the BMC values for the MMP data (Fig. 3F) and previously determined basal oxygen consumption rate (OCR) (van der Stel et al. 2020) (Fig. 3G).

To establish a robust mitochondrial toxicant gene signature, we selected 7 hits from the 23 probes unique for the ETC inhibitor-induced effects upon the mitochondria (Table 1). The selection criterion was a log2FC of > 2 or < − 2 or a direct link to mitochondria, which would facilitate detection when used as biomarker (Suppl. Figure 3G and H). To assess the possible involvement of the selected signature genes in the modulation of mitochondrial toxicity, rotenone treatments were combined with RNA interference. We focused on the signature genes that were upregulated (*CYP3A5, KLHL24, NOS3* or *PFKP*) and used RNA interference to address their role. The depletion of *PFKP* itself resulted in a slight reduction of viability while the other siRNAs had no effect under non-treated conditions (Fig. 3H). *NOS3* depletion led to an increased sensitivity toward rotenone, suggesting that NOS3 is involved in an adaptive response during ETC inhibitor treatment (Fig. 3I). Depletion of the other signature genes did not significantly impact on rotenone-induced cytotoxicity.

**Table 1:** Gene selected from ETC inhibitor screen

Gene	Entrez	Gene	Function	Cellular compartment
Upregulation				
ARHGEF12	23,365	Rho guanine Nucleotide exchange factor 12	*Process initiated by extracellular stimuli*	Cytosol, nucleus
CYP3A5	1577	Cyp enzyme 3A5	*Drug metabolism, synthesis of cholesterol, steroids and other lipids*	Endoplasmic reticulum
FCER1G	2207	Fc fragment of IgE receptor Ig	*Allergic reactions*	Cell membrane
FEZ2	9637	Fasciculation and elongation protein Zeta 2	*Axon bundling and elongation*	Nucleus
ITGB5	3693	Integrin subunit beta 5	*Cell-surface interaction*	Cell membrane
JAK1	3716	Janus kinase 1	*Cytokine signal transduction*	Cytosol, nucleus
KLHL24	54,800	Kelch-like family member 24	*Ubiquitin ligase substrate receptor involved, i.e., in keratin stability*	Cytosol, nucleus
NOS3	4846	Nitric oxide synthase 3	*Production of signaling molecule nitric oxide (NO)*	All compartments
OAS1	4938	2′–5′-Oligoadenylate synthetase 1	*RNA degradation*	Cytosol, nucleus
PFKP	5214	Phosphofructokinase, platelet	*Regulation of glycolysis*	Cytosol
Downregulation				
CDC6	990	Cell division cycle 6	*Essential in DNA replication*	All compartments, except for endoplasmic reticulum, endosomes and lysosomes
COPZ1	22,818	COPI coat complex subunit zeta 1	*Autophagy*	Cytosol, Golgi
DDC	1644	Dopa decarboxylase	*Involved in the production of dopamine, serotonin and tryptamine*	Cytosol
KDELR2	11,014	KDEL endoplasmic reticulum protein retention receptor 2	*Recycling of proteins between Golgi and ER*	Endoplasmic reticulum
MAL2	114,569	Mal, T cell differentiation protein 2	*Protein transport*	Cell membrane
Brp44/ MCP2	25,874	Mitochondrial pyruvate carrier 2	*Pyruvate metabolism, TCA cycle*	Mitochondrion
PEX3	8504	Peroxisomal biogenesis factor 3	*Peroxisome biosynthesis*	Peroxisome, endoplasmic reticulum, nucleus
PRDX3	10,935	Peroxiredoxin 3	*Mitochondrial antioxidant*	Mitochondria
TUBG1	7283	Tubulin gamma 1	*Microtubules formation*	Cytoskeleton
VAPB	9217	VAMP associated protein B And C	*Trafficking*	Endoplasmic reticulum, Golgi
WDR61	80,349	WD repeat domain 61	*Regulation transcription*	Cytosol, nucleus

Table includes information concerning gene name, direction of the transcriptomic change upon ETC inhibitor exposure, full name—function, main location in the cell, organs in which the RNA is present. All gene-specific information was collected using genecard.org (Stelzer et al. 2016)

To address whether this seven-gene signature could be used in a biomarker approach for flagging of possible mitochondrial toxicity liabilities, we evaluated gene expression data for exposure to chemicals known to cause DILI and available for PHH in the TG-GATES database. A list of 156 such chemicals was shortened to focus on 23 chemicals with mitochondrial liabilities in HepG2 cells (Table 2) (Eakins et al. 2016). Four out of the seven selected genes (*CDC6*, *DDC*, *CYP3A5* and *KLHL24*) were similarly affected in PHH as in HepG2 by the chemicals classified as ETC inhibitors (Fig. 3J), except for tamoxifen which did not induce major changes in any gene measured (data not shown). This gene set selected for mitochondrial toxicity via ETC inhibition in HepG2 did not separate DILI compounds with from those without mitochondrial liabilities in PHH (Fig. 3K).

**Table 2:** Selection of DILI compounds

Compound	Mitochondrial target (*)		FDA DILI rank (#)		TG-GATES	Exposure info [µM] (*,**)				
	Mechanism Literature	Predicted mechanism	Severity	DILI Concern		cMax	Eakins	Low	Middle	High
Known mitotoxicant										
Acetaminophen	ETC inhibitor	ETC inhibitor	5	Most-DILI-Concern	APAP	132.25	**5660**	200	1000	5000
Acetylsalicylic acid	ETC inhibitor	Cytotoxicity	NA	NA	No					
Amiodarone	Other	Other	8	Most-DILI-Concern	AM	1.17	**13.4**	0.28	1.4	7
Benzbromarone	Uncoupler	Uncoupler	8	Most-DILI-Concern	BBr	4.36	2.56	4	20	100
Clotrimazole	Substrate inhibitor	Substrate inhibitor	3	Less-DILI-Concern	No					
Diclofenac	Other	Other	8	Most-DILI-Concern	DFNa	6.4	235	16	80	400
Entacapone	Uncoupler	Substrate inhibitor	0	Less-DILI-Concern	No					
Flutamide	ETC inhibitor	ETC inhibitor	8	Most-DILI-Concern	FT	4.59	32.5	2	10	50
Ketoconazole	ETC inhibitor	ETC inhibitor	8	Most-DILI-Concern	KC	7	2.09	0.6	3	15
Mefloquine	ATP synthase	ETC inhibitor	7	Less-DILI-Concern	No					
Menadione	Other	Uncoupler	NA	NA	No					
Metformin	ETC inhibitor	None	0	Less-DILI-Concern	MFM	12.39	**NA**	40	200	1000
Nefazodone	ETC inhibitor	Substrate inhibitor	8	Most-DILI-Concern	NZD	2.61	0.6	1.2	6	30
Nimesulide	Uncoupler	Other	8	Most-DILI-Concern	NIM	18.05	**456**	13.2	66	330
Nitrofurantoin	ETC inhibitor	ETC inhibitor	8	Most-DILI-Concern	NFT	6	48.3	5	25	125
Paraquat	ETC inhibitor	Other	NA	NA	No					
Paroxetine	ETC inhibitor	ETC inhibitor	8	Less-DILI-Concern	No					
Perhexiline	Fatty acid oxidation inhibitor	Substrate inhibitor	8	Most-DILI-Concern	PH	2.16	6.45	0.6	3	15
Phenformin	ETC inhibitor	ETC inhibitor	NA	NA	No					
Pioglitazone	ETC inhibitor	ETC inhibitor	3	Less-DILI-Concern	No					
Primaquine	Uncoupler	None	0	No-DILI-Concern	No					
Promethazine	ETC inhibitor	Cytotoxicity	5	Less-DILI-Concern	No					
Rosiglitazone	ETC inhibitor	ETC inhibitor	3	Less-DILI-Concern	RGZ	0.86	5.81	12	60	300
Tamoxifen	ETC inhibitor	ETC inhibitor	8	Most-DILI-Concern	TMX	1.21	9.58	1	5	25
Tolcapone	Uncoupler	Uncoupler	8	Most-DILI-Concern	No					
Troglitazone	ETC inhibitor	ETC inhibitor	8	Most-DILI-Concern	No					
Unknown mitochondrial liability										
Amitriptyline	None	None	5	Less-DILI-Concern	AMT	0.22	12.7	0.6	3	15
Atenolol	None	None	4	Less-DILI-Concern	No					
Atorvastatin	None	Other	5	Most-DILI-Concern	No					
Azacytidine	None	None	NA	NA	No					
Azathioprine	None	Other	5	Most-DILI-Concern	AZP	1.43	**200**	2.92	14.6	73
Benazepril	None	Cytotoxicity	4	Less-DILI-Concern	No					
Betaine	None	None	0	No-DILI-Concern	No					
Bosentan	None	Cytotoxicity	7	Most-DILI-Concern	No					
Buspirone	None	Substrate inhibitor	3	Ambiguous	BPR	0.01	25.1	1.2	6	30
Chloroquine	None	None	3	Less-DILI-Concern	No					
Chlorpromazine	None	Cytotoxicity	2	Less-DILI-Concern	CPZ	0.61	5.07	0.8	4	20
Clomipramine	None	Substrate inhibitor	8	Most-DILI-Concern	CPM	0.19	**19.2**	0.4	2	10
Clozapine	None	Other	5	Most-DILI-Concern	CZP	0.91	14.2	2	10	50
Fialuridine	None	None	8	Most-DILI-Concern	No					
Flecainide acetate	None	None	7	Less-DILI-Concern	No					
Fluoxetine	None	ETC inhibitor	3	Less-DILI-Concern	No					
Fluvastatin	None	ATP synthase	3	Less-DILI-Concern	No					
Furosemide	None	None	2	Ambiguous	FUR	11.76	500	100	500	2500
Methapyrilene	None	None	NA	NA	MP	115	219	24	120	600
Metoclopramide	None	None	5	Less-DILI-Concern	No					
Mibefradil dihydrochloride	None	Uncoupler	NA	NA	No					
Miconazole	None	ETC inhibitor	NA	NA	No					
Nicardipine	None	None	3	Ambiguous	No					
Nortriptyline	None	None	8	Most-DILI-Concern	No					
Oxybutynin	None	ETC inhibitor	0	No-DILI-Concern	No					
Physostigmine	None	None	0	No-DILI-Concern	No					
Risperidone	None	ETC inhibitor	3	Less-DILI-Concern	No					
Sertraline	None	ETC inhibitor	3	Less-DILI-Concern	No					
Simvastatin	None	ETC inhibitor	3	Less-DILI-Concern	SST	0.02	5.29	1.2	6	30
Streptomycin	None	None	0	No-DILI-Concern	No					
Thioridazine	None	Cytotoxicity	5	Less-DILI-Concern	TRZ	0.55	6.04	0.6	3	15
Verapamil	None	Cytotoxicity	3	Less-DILI-Concern	No					

The DILI compound selection based on Eakins 2016. The subdivision mitochondrial toxicant or not in vivo is kept similar as previously decided by Eakins et al. and is based on their literature search. “Mechanism literature” is the mode of action found in literature. “Predicted mechanism” is the mode of action identified by Eakins et al. using in HepG2. “FDA DILI rank” included the DILI classification as defined by the FDA (#; https://www.fda.gov/science-research/liver-toxicity-knowledge-base-ltkb/drug-induced-liver-injury-rank-dilirank-dataset). “Exposure info [uM]” includes the cMax as defined by Eakins 2016 et al.(*), the lowest effective concentration measured in any of the assays of Eakins et al. (bold = a concentration above the highest concentration tested in TG-GATES) and the three concentrations used for the PHH transcriptomics data in the TG-GATES (** (Igarashi et al. 2015))

In summary, using the S1500 +  + probe set, TempO-Seq could not distinguish active CI from active CIII compounds. However, a gene set was identified whose transcription was specifically affected by mitochondrial toxicants (active CI and CIII inhibitors that affected MMP, ATP production and cell proliferation) and not by other toxicants in HepG2. Gene silencing associated one of these genes, *NOS3*, with adversity in HepG2 cells. A subset of this gene set was also modulated in response to DILI compounds with confirmed ETC inhibitory activity in PHH, but in these cells such a response was also seen with certain compounds not classified as mitochondrial inhibitors.

### Pathway and gene network analysis shows ETC inhibitors trigger responses affecting proliferation, protein homeostasis, and early stress responses

**Fig. 4:**
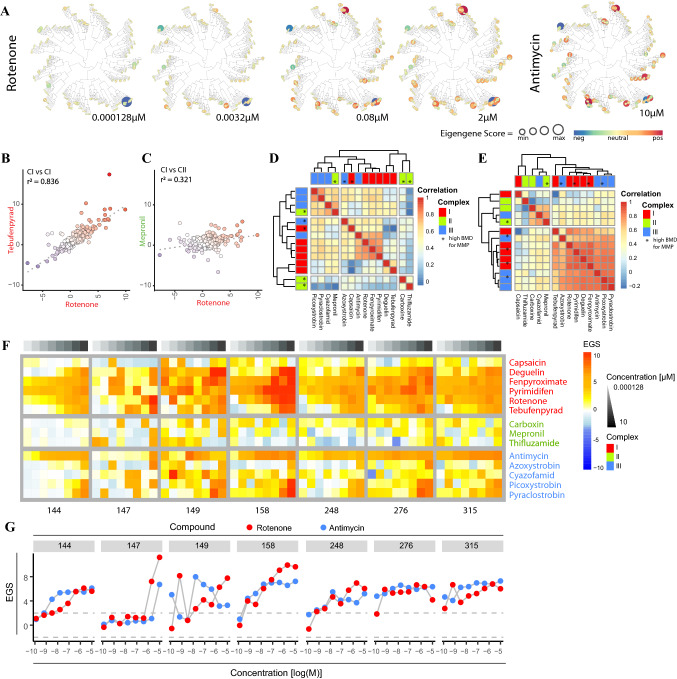
Gene network activation by MRC inhibitors based on human hepatocyte WGCNA datasets. **A** Projection of S1500 +  + TempO-Seq HepG2 expression data on an established WGCNA map of PHH created from TG-GATES data. Four panels show the module EGSs upon 24 h exposure to 4 concentrations of rotenone. Color range from blue (negative EGS) to red (positive EGS) and are resigned per map. The size of the circle corresponds to the absolute EGS per module. **B** and **C** Example correlation plots of the EGS per WGCNA module upon exposure to non-toxic concentrations comparing rotenone (2 µM) to **B** tebufenpyrad (10 µM) or **C** mepronil (10 µM). **D** Correlation plot of all tested ETC inhibitors showing a correlation score based on the EGS scores of all modules per condition. The concentrations selected were a tested concentration above the BMD value of the Rho123 data per treatment (Suppl. Figure 4D). This BMD value was determined using BMDexpress. **E** Correlation plot of all tested ETC inhibitors showing a correlation score based on the EGS scores of all modules per condition. The selected concentration was the highest tested non-toxic concentration per treatment (Suppl. Figure 4D). **F** Heatmap of seven modules showing Eigengene Scores (EGS) upon 24 h exposure to a concentration range of 14 complex inhibitors. The modules were selected based on being upregulated in both the CI and CIII correlation plot (Suppl. Figure 6A). **G** Concentration response curves of Eigen Gene Scores (EGS) per selected module upon 24 h exposure to rotenone or antimycin. The modules were selected based on being upregulated in both the CI and CIII correlation plot

We next moved from the analysis of responses at the individual gene level to analysis of gene networks and pathways, which may enhance translation across model systems. First, we took advantage of previously established weighted gene co-expression network analysis (WGCNA) modules for PHH comprising ~ 400 gene modules that can be visualized as a toxicogenomics map (Callegaro et al. 2021). The ETC inhibitor TempO-Seq transcriptomics data obtained in HepG2 was projected on these prior established co-expression modules and used to calculate module eigengene scores (EGSs) to monitor chemical-induced changes in these modules. Concentration-dependent up- and downmodulation of various modules was observed upon exposure to rotenone and a clear overlap was seen in the affected modules upon exposure to the highest non-toxic concentration of rotenone (2 µM) and antimycin (10 µM) (Fig. 4A). The effects of different ETC inhibitors on module eigengenes were highly correlated within CI (*r*^2^ = 0.836) and CIII inhibitors (*r*^2^ = 0.853), but not within CII inhibitors (*r*^2^ = 0.384) (Fig. 4B, Suppl. Figure 4A and B). Moreover, effects on module eigengenes of CI (rotenone) correlated with the effects of CIII (antimycin) (*r*^2^ = 0.791), but not with the effects of CII inhibition (mepronil) (*r*^2^ = 0.321) (Fig. 4C, Suppl. Figure 4B). In general, the S1500 +  + probe set allowed a module coverage of 30–50% and affected genes per module generally overlapped between the exposure to rotenone and antimycin (bold genes in the table of Suppl. Figure 4C). Comparing all tested chemicals at concentrations just above the BMD of the MMP data did in fact show separation between CI and CIII inhibitors with the exception of antimycin and azoxystrobin that were placed in a CI cluster (Fig. 4D, Suppl. Figure 4D). Of the CII inhibitors, mepronil was placed in the CIII cluster. Increasing exposure to the highest non-cytotoxic concentrations that far exceeded the effective mitochondrial perturbation level and may cause off-target effects did not enhance CI versus CIII separation, but resulted in separation of strong mitotoxicants versus weak/non-mitotoxicants (Fig. 4E, Suppl. Figure 4D). The top ten upregulated modules for CI and III inhibitors included seven common modules (Fig. 4F, G and Suppl. Figure 4C and 5). These modules were associated with oxidative stress (144), signal transduction (149, 158, 248 and 276), metabolism (147, 149 and 248) and transport (149 and 315).

**Fig. 5:**
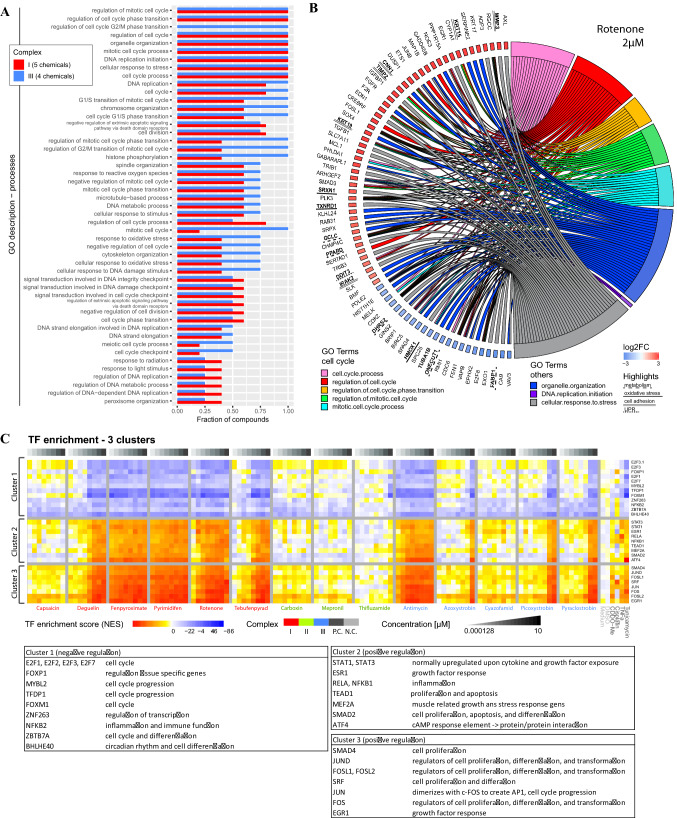
Transcriptional reprogramming after treatment with agrochemical MRC inhibitors. **A** GO enrichment analysis performed using GOrilla software. Genes considered in the analysis demonstrated a padj < 0.05, log2FC < or > 0.58 and *p* value of the William trend test < 0.05. The graph only represents GO terms effected by at least 2 compounds of the CI or CIII inhibitors. The blue bar represents CI inhibitors (deguelin, fenpyroximate, pyrimidifen, rotenone and tebufenpyrad) and the red bar represents CIII inhibitors (antimycin, azoxystrobin, picoxystrobin and pyraclostrobin) **B** Distribution plot of gene expression data upon exposure to 2 µM rotenone. The genes are distributed over the eight GO terms affected by all included CI and CIII inhibitors. Only the genes having a log2FC < − 2 or < 2 are visualized. **C** Heatmap of three transcription factor enrichment clusters showing normalized enrichment score (NES) upon 24 h exposure to concentration range of 14 complex inhibitors, 4 positive controls (P.C.) (60 nM CDDO-me, 1 mg/ml cisplatin, 10 ng/mL TNFα and 12 µM tunicamycin) and vehicle control (N.C.). Complete cluster of all transcription factors are shown in Suppl. Figure 4. Transcription factor enrichment study was based on the transcription factor fingerprint in the DoRothEA database and using the viper package for the enrichment assessment. The used confidence parameter in the DoRothEA data set was ABC. Below heatmap are three tables including the transcription factors from the three clusters plus biological function collected from genecard (https://www.genecards.org/)

We subsequently analyzed GO term enrichment to explore pathways triggered by ETC inhibition. In agreement with the connection to cell proliferation (see Fig. 2F, G), GO term enrichment analysis demonstrated enrichment for perturbation of cell cycle related pathways upon exposure to all CI and III inhibitors (Fig. 5A), as expected in proliferating HepG2 cells. Other GO terms regulated by all CI and III inhibitors included responses to stress, organelle organization and DNA replication. The top up- and down regulated genes for these biological processes were mapped in Fig. 5B. The response to stress was captured by changes in metabolism-related genes (*GCLC, PPARD, DHRS2, ONECUT1, FABP1*) and induction of oxidative stress responders (*SRXN1, TXNRD1* and *HMOX1*). The GO term organelle organization was mostly driven by changes in the expression of genes involved in cell adhesion and matrix interaction (including amongst others *KRT, CNN1, IRAK2, TUBA1B, MMP3, TIMP2*). The cell cycle term comprised factors involved in the cell cycle itself (including e.g., *RGCC, DUSP1, PLK3, CDK2, CDC6*), growth factor responses (*IGFBP1, EGFR, FOSL1, TFGB1* and *SMAD3*), DNA replication and related replication control (*GADD54B, POLE2, BRIP1, RMI1* and *FEN*1) and regulation of cell death and differentiation (including *SOX4, MCL1, TRIB3, BMF, BRIC5*).

Our targeted TempO-Seq probe set was biased toward pathways important in toxicology, which could result in an increased threshold for identification of toxicity-related pathways. We next used transcription factor (TF) enrichment analysis, which combines the target gene expression profiles for all measured probes per TF and considers the overall direction of the response, rendering the enrichment less dependent on the number of probes assessed. Again, TF profiles demonstrated a concentration-dependent modulation by CI and CIII inhibitors, but not CII ETC inhibitors (Suppl. Figure 6). Clustering of the enriched TFs resulted in three clusters demonstrating a clear concentration dependency based on visual inspection (Fig. 5C and Suppl. Figure 6). These three TFs clusters belonged to three major biological responses: a cluster of downregulated TFs mostly involved in proliferation; a cluster of upregulated TFs involved in inflammation and protein homeostasis; and a cluster of upregulated TFs involved in early stress responses (Fig. 5C).

### Combining TempO-Seq and high-content imaging indicates that amino acid response (AAR) is triggered by complex I and III ETC inhibitors

**Fig. 6:**
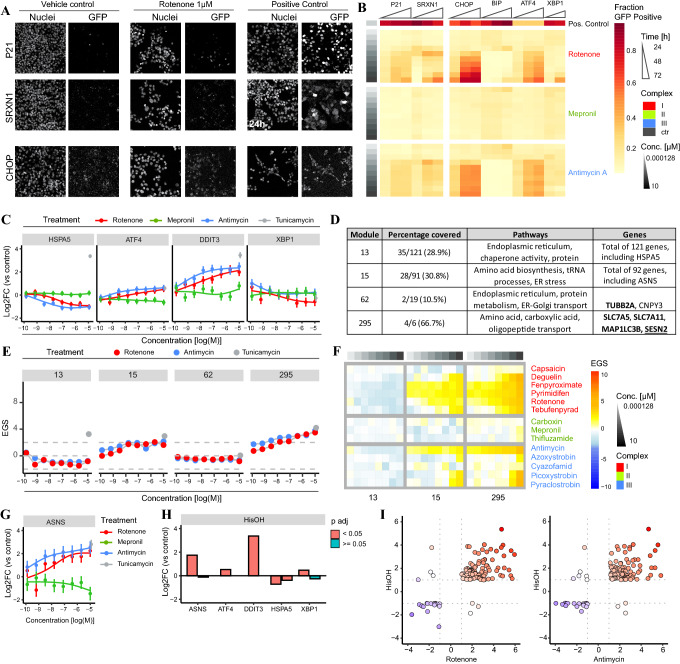
Effects of various agrochemical MRC inhibitors on cellular stress activation: high-content imaging and transcriptomics analysis. **A** Representative pictures of Hoechst and GFP (CHOP-GFP, P21-GFP and SRXN1-GFP) upon 24 or 72 h exposure to the vehicle control, rotenone 1 µM and the positive controls (6 µM tunicamycin of the CHOP-GFP reporter, 25 µM etoposide for the P21-GFP reporter and 0.1 µM DEM for the SRXN1-GFP reporter). **B** Heatmap of fraction GFP-positive cells for P21-GFP, SRXN1-GFP, CHOP-GFP, BIP-GFP, ATF4-GFP, and XBP1-GFP upon exposure to a concentration range of rotenone, mepronil and antimycin at 24, 48 and 72 h. Positive control compounds included 6 µM tunicamycin of the CHOP-, BIP-, ATF4- and XBP1-GFP reporter, 25 µM etoposide for the P21-GFP reporter and 0.1 µM diethyl maleate (DEM) for the SRXN1-GFP reporter. Cells considered as GFP positive demonstrated an integrated GFP intensity of two times the vehicle control. Values are a mean of two or three biological replicates. **C** Concentration response curves of the log2FC ± SE for HSPA5, ATF4, DDIT3 and XBP1 upon exposure to rotenone, mepronil or antimycin. The log2FC value of tunicamycin 6 µM is represented as a gray dot. **D** Table of module 13, 15, 62 and 295 describing: the percentage coverage when projecting the S1500 +  +  + set, pathway enriched pathways of this specific module and the genes measured in that particular module (bold: log2FC > 0.58 upon exposure to 2 µM rotenone and 10 µM antimycin, underlined: hub gene of this module). The modules were selected based on their involvement in the ER stress response (Callegaro et al. 2021). **E** Concentration response curves of eigengene scores (EGS) per selected module upon 24 h exposure to rotenone, antimycin or tunicamycin. **F** Heatmap of three modules showing Eigengene Scores (EGS) upon 24 h exposure to a concentration range of 14 complex inhibitors. The modules were selected based on being upregulated in both the CI and CIII correlation plot (Suppl. Figure 6A) or being identified as ER module by Callegaro et al. (2021). **G** Concentration response curves of the log2FC ± SE for ASNS upon exposure to rotenone, mepronil or antimycin. The log2FC value of tunicamycin 6 µM is represented as a gray dot. **H** log2FC for ASNS, ATF4, DDIT3, HSPA5, XBP1 upon exposure to 5 mM HisOH for 4 h (Shan 2010). Multiple bars per gene represent single microarray probes. **I** Correlation plots of HepG2 cells exposed to 5 mM HisOH for 4 h (Shan 2010) and non-toxic concentration of rotenone (2 µM) or antimycin A (10 µM) for 24 h. Genes are included with a log2F < − 0.58 or > 0.58 and a p-adjusted value below 0.05

To allow dynamic monitoring of the effects on cell cycle progression, protein homeostasis, and stress responses predicted by the gene- and pathway analyses mentioned above, the expression of P21 (cell cycle arrest), SRXN1 (oxydative stress), and ATF4 and CHOP (ER stress) was monitored using GFP reporter cell lines generated using BAC technology (Wink et al 2014). Activation of P21 and SRXN1 was minimal for all inhibitors, but clearly upregulated upon exposure to the positive controls (respectively, etoposide and DEM) (Fig. 6A, B, Suppl. Figure 7). Exposure to both CI and CIII inhibitors, but not CII inhibitors, resulted in a concentration-dependent increase in ATF4 (Figs. 5C and 6B, Suppl. Figure 7). The transcription factor ATF4 is involved in protein homeostasis in the endoplasmic reticulum (ER) as well as in mitochondria (Hetz 2012, Melber and Haynes 2018). To further explore the possible perturbation of protein homeostasis and the induction of the related unfolded protein response (UPR), GFP reporters for BIP (*HSPA5*), CHOP (*DDIT3*) and XBP1 were monitored. Interestingly, XBP1, which is downstream of the UPR sensor IRE1α, was unaffected by ETC inhibition at all non-toxic concentrations. Moreover, while CHOP was upregulated, BIP, a molecular chaperone in the ER was downregulated further arguing against the induction of a UPR (Fig. 6A, B, Suppl. Figure 7). Overall, the concentration-dependent changes observed with GFP reporters corroborated the TempO-Seq data (Fig. 6C).

Revisiting four ER stress-related WGCNA modules (13, 15, 62 and 295; (Callegaro et al. 2021)), in the PHH data mentioned above (Fig. 4), supported the observed difference between the UPR stress inducer tunicamycin and the ETC inhibitors, i.e., deactivation of module 13 (including HSPA5) and activation of module 15 (including ASNS) was observed (Fig. 6D–F). The absence of an expected upregulation of module 62 was most likely caused by the lower coverage when using the s1500 +  + gene set (10.5%). Interestingly, modules 15 and 295 are highly enriched for amino acid biosynthesis and transport. The absence of an effect on XBP1 and the opposite direction of the response for HSPA5/BIP and ATF4 did not support UPR activation, but may instead point to nutrient (amino acid) deprivation, which also leads to ATF4 upregulation (Krall et al. 2021, Ye et al. 2010). Indeed, mRNA of the ATF4 target gene *ASNS*, encoding an enzyme responsible for aspartate to asparagine conversion that is upregulated upon asparagine shortage, showed a fourfold increase upon exposure to rotenone and antimycin (Fig. 6G).

To explore similarities between responses to mitochondrial toxicants and amino acid deprivation, we analyzed previously published gene expression data for HepG2 exposure to the histidinyl tRNA synthetase inhibitor (HisOH) (Shan et al. 2010). HisOH simulates amino acid deprivation and activates a response to amino acid starvation, termed the amino acid response (AAR). HisOH exposure resulted in a similar expression pattern of *ATF4* (upregulation) and *HSPA5*/BIP (downregulation) as observed upon rotenone and antimycin exposure (Fig. 6H). In addition, comparisons of the effect on HepG2 RNA expression assessed using microarrays for HisOH (Shan et al. 2010) and rotenone and antimycin (s1500 +  + ; our data) demonstrated strikingly similar directionality with only seven to eight genes exhibiting an opposite effect upon exposure (Fig. 6I).

**Fig. 7:**
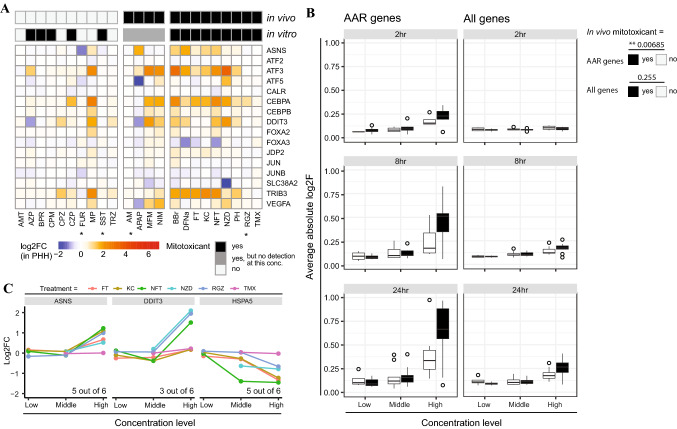
Extrapolation of electron transport chain (ETC) inhibitor markers to PHH. **A** Heatmap of 16 genes involved in the amino acid response (Shan 2010). The presented values are the log2FC values from the TG-GATES database for the selected genes set upon 24 h exposure to highest concentration available for DILI compounds classified by Eakins 2016 (Table 2) (* = exceptions are with middle concentration, because the highest concentration was more than 100-fold the cMax value). **B** Box plot of the average of the absolute log2FC values of AAR-related genes versus “all genes” for all DILI compounds with versus without in vivo mitotoxicant activity at 2, 8 and 24 h exposure to the low, middle, and high concentration. P values were obtained using a generalized linear model of the average abs log2F addressing the following variables: yes/no in vivo mitochondrial toxicant; concentration; time. **C** Log2FC data from the TG-GATES database for ASNS, DDIT3 and HSPA5 in PHH upon 24 h exposure to three concentrations of DILI-inducing compounds known to also effect the ETC (Table 2)

Lastly, to further evaluate the AAR in the context of mitotoxicants in PHH, expression of genes previously shown to be involved in AAR (Shan et al. 2010) was evaluated in the TG-GATES PHH data for DILI compounds previously assessed for mitochondrial perturbing potential (Table 2, Fig. 7A) (Eakins et al. 2016). A large proportion of DILI compounds with confirmed in vivo and in vitro mitotoxic activity affected several of the AAR-related genes. On the other hand, most DILI compounds lacking in vivo mitotoxic activity (some of which had also been associated with in vitro mitotoxicity) did not affect AAR-related genes. Methapyrilene was a noticeable exception in this group, affecting ~ half of the AAR-related genes. Generalized linear regression modeling showed time- and concentration-dependent increase in the absolute log2FC for “AAR genes” (time, *p* = 3.74e−05; concentration, *p* = 3.88e−16) and “all genes” (time, *p* = 1.92e−07; concentration, *p* = 5.64e−14) for confirmed in vivo mitotoxicitants and non-mitotoxicants. However, while the average of the absolute log2FC of the AAR gene set was significantly higher (*p* = 0.00685) for in vivo mitotoxicants as compared to the other chemicals at 2, 8, and 24 h exposure (Fig. 7B; AAR genes), no difference between in vivo mitotoxicants and other chemicals was observed for “all genes” in the microarray (Fig. 7B; all genes). This suggested that the AAR represents an early response to mitotoxic insults for DILI compounds in both HepG2 and PHH. In agreement, exposure to ETC inhibiting DILI compounds in most cases affected the expression of genes at the bifurcation between UPR and AAR (*ASNS*, *DDIT3*/CHOP and *HSPA5*/BIP) with similar directionality in PHH as observed upon mitotoxicant exposure in HepG2, further corroborating an AAR in response to ETC inhibition (Fig. 7C).

Together, these findings, indicate that active ETC inhibitors trigger cellular signaling pathways including those involved in the evolutionary conserved response to amino acid starvation, AAR. This response appears to be shared between HepG2 and PHH.

## Discussion

In this study, we have systematically assessed the perturbation of mitochondrial functioning by a diverse set of agrochemicals that target the electron transport chain (ETC) CI, CII and CIII and its subsequent cellular consequences. Our results indicate that CI inhibiting agrochemicals are most potent as mitotoxicants, followed by CIII inhibitors, while C II inhibitors hardly have any effect. High throughput transcriptomics approaches were identified to be at least as sensitive as mitochondrial integrity and OCR measurements to define biological perturbations by ETC inhibitors. The observed transcriptional alterations demonstrated ETC inhibition related effects rather than complex inhibitor specific effects. Our data indicate that integration of high-content imaging approaches and high throughput transcriptomics technology is a powerful approach to provide mechanistic weight of evidence to support hazard evaluation of mitochondrial toxicants for full safety assessment.

The majority of studies assessing mitochondrial perturbation in high throughput focus on a few early time points using direct mitochondrial-related endpoints to identify potential mitochondrial toxicants (Shah et al. 2016, Wills et al. 2015, Hallinger et al. 2020, Xia et al. 2018, Attene-Ramos et al. 2015). In contrast, we studied a combination of dynamic imaging-based measurements of mitochondrial functioning and cellular stress signaling responses to unravel the relationship between mitochondrial perturbation and cellular toxicity. By employing a high-content live cell imaging set-up, the temporal dynamics of mitochondrial perturbation were determined by measuring MMP. Assessment of the MMP dynamics upon exposure to a set of mitochondrial ETC inhibitors demonstrated an early onset of concentration-dependent MMP depletion for most CI and CIII inhibitors, delayed perturbation after hydra-methylnon exposure and no/minimal MMP disruption for the CII inhibitors. This indicates direct interaction for most CI and CIII inhibitors and indirect targeting of the mitochondria for hydramethylnon. Evaluation of the estimated decay time and maximal MMP reduction did not lead to a clear separation of CI and CIII inhibitors. Nevertheless, these parameters can be used to study correlations to other chemical properties, as was demonstrated by the positive correlation to the logP value. Altogether, these findings illustrate added value of using image-based temporal measurements of mitochondrial functioning to assess mitochondrial perturbation.

The cellular outcome after mitochondrial perturbation depends not only on the strength of the perturbation, but also on the cellular capacity to adapt to this (partial) loss of mitochondrial function. For this purpose, the imaging-based assessment of mitochondrial perturbation was combined with a transcriptomic readout focusing on a broad set of toxicology-related transcripts (Waldmann et al. 2014, Limonciel et al. 2018). Exposure to both CI and CIII inhibitors triggered major and nearly identical changes in this set of transcripts in a concentration-dependent manner, indicating a generic response upon ETC inhibition instead of complex specific responses. Previous studies have shown that mitochondrial-targeting chemicals can be clustered based on expression profiles of mRNA subsets (Pearson et al. 2016, Simon et al. 2019). Our broad set of transcripts was used to study the classification of specific and potent CI and CIII inhibitors based on cellular responses upon mitochondrial perturbation. This led to the identification of an ETC inhibition specific gene set that included *CYP3A5, KLHL24, NOS3, PFKP, CDC6, DDC*, and *MPC2*. We verified that these genes were not affected by chemicals well known to induce stress responses, other than ETC inhibition related responses, including DNA damage, reactive oxygen species, unfolded proteins and inflammation. Moreover, the BMC for the onset of gene expression changes was overall lower than the BMC for MMP depletion or OCR decrease. For *CYP3A5, CDC6* and *DDC* no direct link to mitochondrial perturbation has been reported so far. On the other hand, downregulation of *PFKP,* involved in glycolysis, has been reported to result in decreased cellular viability when combined with mitochondrial toxicants (To et al. 2019). *KLHL24* and *NOS3* are involved in fatty acid metabolism^3^ and mitochondria biogenesis, respectively (Nisoli and Carruba 2006, Nisoli et al. 2004). Inhibition of *MCP2*, a mitochondrial pyruvate transporter, is reported to result in upregulation of aerobic glycolysis (Li et al. 2017, Schell et al. 2014). We anticipate that the genes in the ETC inhibition specific set are part of an adaptive response where cells switch from oxidative phosphorylation to glycolysis, increase their fatty acid metabolism and support the production of new mitochondria. Indeed, in particular NOS3 depletion increased the susceptibility of the HepG2 cells to CI inhibition. This gene set may serve as a biomarker panel to flag chemicals and drugs with ETC perturbing potential in early phase high throughput screening using HepG2. However, although regulation by mitotoxicants of this set is confirmed in PHHs, these genes are also sensitive to non-mitotoxicants in this system and can therefore not support mechanistic studies toward the mode of action of various chemicals in PHHs.

Translation of gene expression markers amongst various in vitro models has been proven to be difficult for various liver models (Boess et al. 2003). Nonetheless, we assume that changes in the expression of groups of genes related to the mode of action of toxicants can be detected across multiple models. The secondary adaptation response will differ depending on the nature of the model being primary material or cell line, including for example the glycolytic capacity of the model in case of mitochondrial toxicants. Toxicity assessment in a tiered testing strategy may start with simple models for larger-scale screening to flag toxicants based on model specific markers. Subsequent steps may use HepG2 having a more mitochondria-dependent phenotype and enhanced metabolic activity (van der Stel et al. 2020, Hiemstra et al. 2019, Ramaiahgari et al. 2014, Boon et al. 2020) by using 3D cell cultures or improved medium or the use of HepaRG cells having higher levels of metabolism (Gerets et al. 2012).

To gain more quantitative insights into cellular signaling triggered by ETC inhibition and to increase the possibility for translation across in vitro models, enrichment of pathways and gene networks was explored. Interestingly, for both CI and CIII inhibitors, the mRNA expression changed for components of the cellular response associated with amino acid deprivation and related responses (AAR) (Krall et al. 2021, Ye et al. 2010, Shan et al. 2010). Analysis of HepG2 BAC HSPA5/BIP-GFP, ATF4-GFP and CHOP/DDIT3-GFP reporters confirmed the observed mRNA expression changes at the protein level. In addition, the gene encoding an aspartate conversion enzyme that is controlled by the ATF4 transcription factor in the AAR, *ASNS*, is induced after ETC CI or CIII inhibition. Pathway analysis showed, as expected for amino acid deprivation, significant changes in the cell cycle, which was in line with the attenuation of proliferation detected by high-content imaging. To strengthen the evidence concerning amino acid deprivation and to support identification of additional ETC inhibition induced biology, PHH-specific weighted co-regulated gene networks were used, allowing the study of cellular adaptation outside the known biological context (Callegaro et al. 2021, Sutherland et al. 2018). The projection of our HepG2 transcription data on this PHH network identified co-regulated gene modules that were affected by mitochondrial ETC perturbation. GO terms related to these modules, as anticipated, included metabolism, oxidative stress responses and mitochondrial trafficking. In addition, assessment of endoplasmic reticulum (ER) stress-enriched modules, specifically describing amino acid processes, corroborate the disturbance of protein homeostasis in those cells as well. Moreover, we find that genes known to be involved in the AAR, in general, are also affected in PHH upon exposure to DILI compounds with mitochondrial liability in vivo. This indicates that AAR may represent a conserved response to ETC inhibition. This observed metabolic adaptation has also been observed in neurons upon mitochondrial toxicity caused by exposure to MMP + or by mutations in *PINK1* and *PARKIN* (Krug et al. 2014, Celardo et al. 2017). In addition, ATF4 and CHOP signaling without a clear UPR response has been observed in the context of muscle disorder (Kaspar et al. 2021). Notably, the AAR is also linked to the “integrated stress response”, that restores balance after amino acid starvation and other types of cellular stress and involves ATF4 (Costa-Mattioli and Walter 2020).

Using the ETC inhibitors, we aimed to identify mitochondrial-specific markers. The identification of ETC inhibition-relevant genes and pathways upon exposure to various drug-induced liver injury (DILI) compounds indicates the important role of mitochondria in the occurrence of DILI. In this study, only mitochondrial ETC inhibitors for transcriptomics analysis have been used, hence an extension of the chemical set is required to evaluate whether other chemicals, targeting mitochondrial citric acid cycle metabolism, ion homeostasis and mitochondrial coupling or mitophagy, induce a similar generic mitochondrial stress response. Transcriptomic profiling supports besides mitochondrial toxicity marker identification also chemical read across. The two rotenoid CI inhibitors in this study, deguelin and rotenone, exemplify a transcriptomics-based read across approach. Gene co-regulation analysis can strengthen chemical read across by clustering based on similar module activity, which is less dependent on the expression level of single genes (Joseph 2017, Serra et al. 2020). Furthermore, the overall change in the transcript profiles demonstrates differences in potency between the two rotenoids and the overlap in pathway enrichment, transcription factor activation and gene network module regulation, underlining the similarities in the ETC inhibitor mode of action. To allow the integration of mode of action information and, with that, the identification of mitochondrial perturbation as primary or secondary event in any form of organ toxicity, it is important to enlarge the range of time points used to identify markers or to perform a biologically driven read across.

To summarize, the mechanisms underlying chemical-induced mitochondrial perturbation and cellular signaling were studied using high-content imaging and targeted transcriptomics. Both technologies are demonstrated to be suitable to qualify and quantify the effects of ETC inhibitors on mitochondrial and cellular signaling dynamics. By employing pathway and gene network analyses evidence is provided for a response to ETC inhibition that involves the AAR. We envision a tiered testing strategy where high-content imaging would identify mitochondrial perturbing chemicals, followed by targeted transcriptomic analysis which identifies subsequent cellular outcomes. Such an approach can provide mechanistic weight of evidence to support hazard evaluation of mitochondrial toxicants.

## Supplementary Information

Below is the link to the electronic supplementary material.

Supplementary Fig. 1: Generation and characterization of cytosolic and mitochondrial ATP biosensors in HepG2 cells. A) Representative pictures to demonstrate cytoplasmic and mitochondrial localization of respectively ateam1.03 and mitAt1.03. B) Representative pictures of the ATP biosensor in the cytoplasm (normal and mutated) and mitochondria upon 2 h exposure to vehicle control or 0.5 µM rotenone. C) Representative images of the Hs578T cell line containing CFP and YFP fluorophores used to adjust imaging settings. D) Representative pictures of ATP-biosensor in HepG2 the cytoplasm plus example Ilastik segmentation of the area used for signal quantification. E) Quantification of 2nd replicate of the ateam1.03 (cytoplasmic) and mitAt1.03 (mitochondrial) upon exposure to 4 concentrations of rotenone or antimycin. F) Representation of the exposure schedule for the ATPlite assay including cell membrane lyses step. Plus schematic representation of change in signal of Rho123 or calcein-AM upon addition of digitonin G) Representative pictures of the Rho123 signal upon addition of 150 µM digitonin (digitonin addition at 0 s). H) Representative pictures of the calcein-AM signal upon addition of only buffer (buffer addition at 0 s). I) Representative pictures of the calcein-AM signal upon addition of 150 µM digitonin (digitonin addition at 0 s). Supplementary Fig. 2: Phenomological model fit of mitochondrial membrane potential dynamics. Representative fit of the Rho123 intensity over time upon exposure to 10 concentrations of antimycin A using the phenomological model. Light grey lines represent data of individual biological replicates, dotted line represents the mean of the 4 biological replicates and the cyan line represents the fitted data. Supplementary Fig. 3: Predictive gene expression profiles for MRC inhibition. A) Concentration response curve of number of DEGs up or down regulated at 24 h exposure per treatment. DEGs are considered when padj < 0.05, log2FC > or < 0.58 and the p-values of the Williams trend test < 0.05. Samples were exposed to a concentration range of rotenone, tebufenpyrad, carboxin, mepronil, antimycin or picoxystrobin (concentration range from 0.000128 µM to 10 µM, with steps of factor 5). B) Correlation plot comparing MMP to number of DEGs after 24 h exposure to a concentration range of rotenone, tebufenpyrad, carboxin, mepronil, antimycin or picoxystrobin (concentration range from 0.000128 µM-10 µM). Values are geometric (MMP) or mean (count) of 4 biological replicates. C) Accumulation curves of best BMD values at 24 h exposure to rotenone, tebufenpyrad, carboxin, mepronil, antimycin or picoxystrobin calculated per probe using the BMDexpress software. Probes were considered when the p-value of the William trend test < 0.05 (dotted line represents the BMD value of the MMP readout at 24 h). D) Plot based on all the gene expression data of all mitochondrial complex inhibitors and separating all genes per individual treatment. Horizontal bars at the left represent the total number of DEGs per treatment which meet the filtering criteria. The vertical bars representing the various group of probes only include groups of more than 6 genes. Blue vertical bar represents the groups of genes affected by all active ETC inhibitors. Grey compound names represent the inactive ETC inhibitors. E) Subset of 302 probes from Fig. 6C separated per CI inhibitor. The plot only represents groups of more than 3 genes. F) Subset of 193 probes from Fig. 6C separated per CIII inhibitor. The plot only represents groups of more than 3 genes. G and H) Validation using real time PCR of 3 down (G) and 4 up (H) regulated hits upon exposure to vehicle control, 6 complex inhibitors (2 µM) and the positive adaptive stress response controls (60 nM CDDO-me, 1 mg/ml cisplatin, 10 ng/mL TNFα and 12 µM tunicamycin). Data is represented as mean of 3 biological replicates ± SD. Supplementary Fig. 4: Gene network activation by MRC inhibitors based on human hepatocyte WGCNA datasets. A) Correlation plot of the EGS per module comparing upon exposure to a non-toxic concentration (10 µM, except for rotenone it is 2 µM) of 2 representative chemicals of CI, CII or CIII inhibitor. The top modules up and down regulated per correlation are labeled B) Correlation plot of the EGS per module comparing upon exposure to a non-toxic concentration (10 µM, except for rotenone it is 2 µM) of rotenone (CI) to mepronil (CII) or antimycin (CIII). The top modules up and down regulated per correlation are labeled. C) Table of module 144, 147, 149, 158, 248, 276 and 315 describing: the percentage coverage when projecting the S1500 +  +  + set, pathway enriched pathways of this specific module and the genes measured in that particular module (bold: log2FC > 0.58 upon exposure to 2 µM rotenone and 10 µM antimycin, underlined: hub gene of this module).D) Table depicting the concentration selected in the heatmaps represented in Fig. 4D and E. Asterisk denotes the chemicals with high BMD values for MMP perturbation. Supplementary Fig. 5: Gene network activation by MRC inhibitors based on human hepatocyte WGCNA datasets. A clustered heatmap (Pearson correlation) of WGCNA module showing Eigen Gene Scores (EGS) upon 24 h exposure to concentration range of 14 complex inhibitors and 4 positive controls (60 nM CDDO-me, 1 mg/ml cisplatin, 10 ng/mL TNFα and 12 µM tunicamycin). The included modules do have an EGS of at least 2 in one of the conditions. Supplementary Fig. 6: Transcriptional reprogramming after treatment with agrochemical MRC inhibitors. A clustered heatmap (based on Pearson correlation) of a transcription factor enrichment study showing normalized enrichment score (NES) upon 24 h exposure to concentration range of 14 complex inhibitors and 4 positive controls (60 nM CDDO-me, 1 mg/ml cisplatin, 10 ng/mL TNFα and 12 µM tunicamycin). Transcription factor enrichment study was based on the transcription factor fingerprint in the DoRothEA database and using the viper package for the enrichment assessment. The used confidence parameter in the DoRothEA data set was ABC. Supplementary Fig. 7: Effects of various agrochemical MRC inhibitors on cellular stress response reporter activation. A) Heatmap of fraction GFP-positive cells for CHOP-GFP, P21-GFP and SRXN1-GFP upon exposure to a concentration range of 22 mitochondrial complex inhibitors at 24, 48 and 72 h. Positive control compounds included 6 µM tunicamycin of the CHOP-GFP reporter, 25 µM etoposide for the P21-GFP reporter and 0.1 µM DEM for the SRXN1-GFP reporter. Cells considered as GFP positive demonstrated an integrated GFP-intensity of two times the vehicle control. Values are a mean of 2 or 3 biological replicates. B) Heatmap of fraction GFP-positive cells for BIP-GFP, ATF4-GFP, CHOP-GFP and XBP1-GFP upon exposure to a concentration range of 22 mitochondrial complex inhibitors at 24, 48 and 72 h. Positive control compounds included 6 µM tunicamycin. Cells considered as GFP positive demonstrated an integrated GFP-intensity of two times the vehicle control. Values are a mean of 2 or 3 biological replicates (PDF 15292 KB)

Supplementary file2 (DOCX 15 KB)

Supplementary file3 (XLSX 151 KB)

## Data Availability

The data generated during the current study are available from the corresponding author on reasonable request.

## Abbreviations

2DG: 2-Deoxy glucose
ADP: Adenosine diphosphate
AIC: Akaike information criterion
ATP: Adenosine triphosphate
BAC: Bacterial artificial chromosome
BMC: Benchmark concentration
cDNA: Complementary DNA
CFP: Cyan fluorescent protein
cMax: Maximal concentration in plasma
DDR: DNA damage response
DEG: Differential expressed genes/probes
DEM: Diethyl maleate
DILI: Drug-induced liver injury
DMEM: Dulbecco’s modified Eagle’s medium
DMSO: Dimethyl sulfoxide
DNA: Deoxyribonucleic acid
CDDO-me: Bardoxolone methyl
EC50: Half-maximal effective concentration
EGS: Eigengene Score
ETC: Electron transport chain
FBS: Fetal bovine serum
FC: Fold change
FRET: Fluorescence resonance energy transfer
Gal: Galactose
GFP: Green fluorescent protein
Glu: Glucose
MMP: Mitochondrial membrane potential
GO: Gene Ontology
N.C.: Negative control
NES: Normalized enrichment score
O/N: Overnight
OCR: Oxygen consumption rate
OSR: Oxidative stress response
OXPHOS: Oxidative phosphorylation
padj: Adjusted *p* value
PBS: Phosphate-buffered Saline
P.C.: Positive control
PCR: Polymerase chain reaction
PI: Propidium iodide
PenStrep: Penicillin–Streptomycin
PHH: Primary human hepatocytes
PVDF: Polyvinylidene difluoride
Rho123: Rhodamine123
RNA: Ribonucleic acid
ROS: Reactive oxygen species
RT: Room temperature
SD: Standard deviation
siRNA: Small interfering RNA
TF: Transcription factor
TG-GATES: Toxicogenomics Project-Genomics Assisted Toxicity Evaluation System
TNFa: Tumor necrosis factor alpha
TP: Time point
UPR: Unfolded protein response
WGCNA: Weighted gene correlation network analysis
YFP: Yellow fluorescent protein

## Footnotes

1 http://brainarray.mbni.med.umich.edu/Brainarray/Database/CustomCDF/genomic_curated_CDF.asp, HGU133Plus2 array version, visited 28-6-2021.

2 https://github.com/mcs07/PubChemPy.

3 https://amp.pharm.mssm.edu/archs4/gene/KLHL24.
