# Supplementary material for: Mapping the cellular response to electron transport chain inhibitors reveals selective signaling networks triggered by mitochondrial perturbation
Source: Arch Toxicol. 2021 Oct 13;96(1):259–85. doi: 10.1007/s00204-021-03160-7 (PMC8748354; doi:10.1007/s00204-021-03160-7)
Supplement: Supplementary file 1 — Supplementary Fig. 1: Generation and characterization of cytosolic and mitochondrial ATP biosensors in HepG2 cells. A) Representative pictures to demonstrate cytoplasmic and mitochondrial localization of respectively ateam1.03 and mitAt1.03. B) Representative pictures of the ATP biosensor in the cytoplasm (normal and mutated) and mitochondria upon 2 h exposure to vehicle control or 0.5 µM rotenone. C) Representative images of the Hs578T cell line containing CFP and YFP fluorophores used to adjust imaging settings. D) Representative pictures of ATP-biosensor in HepG2 the cytoplasm plus example Ilastik segmentation of the area used for signal quantification. E) Quantification of 2nd replicate of the ateam1.03 (cytoplasmic) and mitAt1.03 (mitochondrial) upon exposure to 4 concentrations of rotenone or antimycin. F) Representation of the exposure schedule for the ATPlite assay including cell membrane lyses step. Plus schematic representation of change in signal of Rho123 or calcein-AM upon addition of digitonin G) Representative pictures of the Rho123 signal upon addition of 150 µM digitonin (digitonin addition at 0 s). H) Representative pictures of the calcein-AM signal upon addition of only buffer (buffer addition at 0 s). I) Representative pictures of the calcein-AM signal upon addition of 150 µM digitonin (digitonin addition at 0 s). Supplementary Fig. 2: Phenomological model fit of mitochondrial membrane potential dynamics. Representative fit of the Rho123 intensity over time upon exposure to 10 concentrations of antimycin A using the phenomological model. Light grey lines represent data of individual biological replicates, dotted line represents the mean of the 4 biological replicates and the cyan line represents the fitted data. Supplementary Fig. 3: Predictive gene expression profiles for MRC inhibition. A) Concentration response curve of number of DEGs up or down regulated at 24 h exposure per treatment. DEGs are considered when padj < 0.05, log2FC > or < 0.58 [file 204_2021_3160_MOESM1_ESM.pdf]

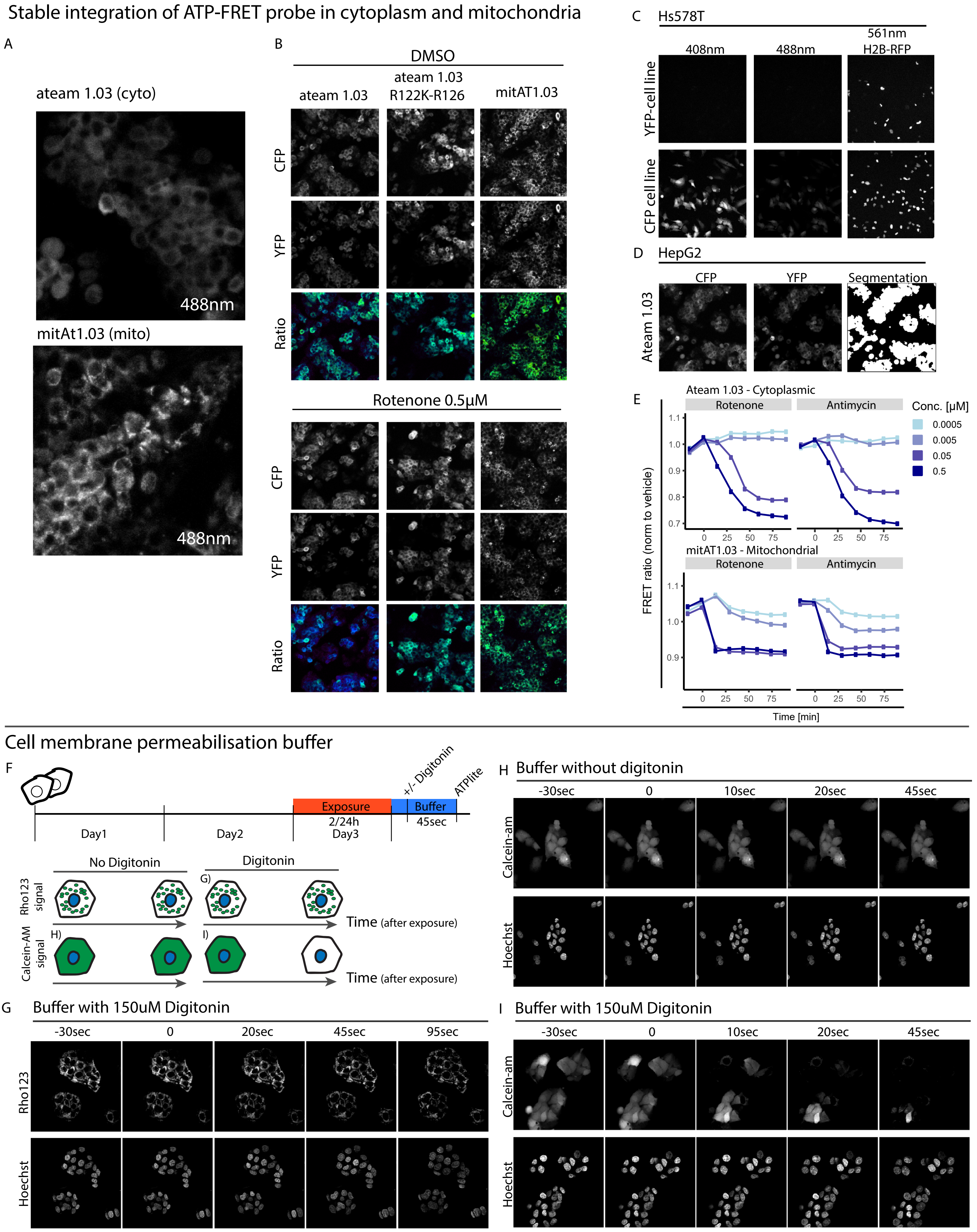

Supplementary figure 1: Generation and characterization of cytosolic and mitochondrial ATP-biosensors in HepG2 cells

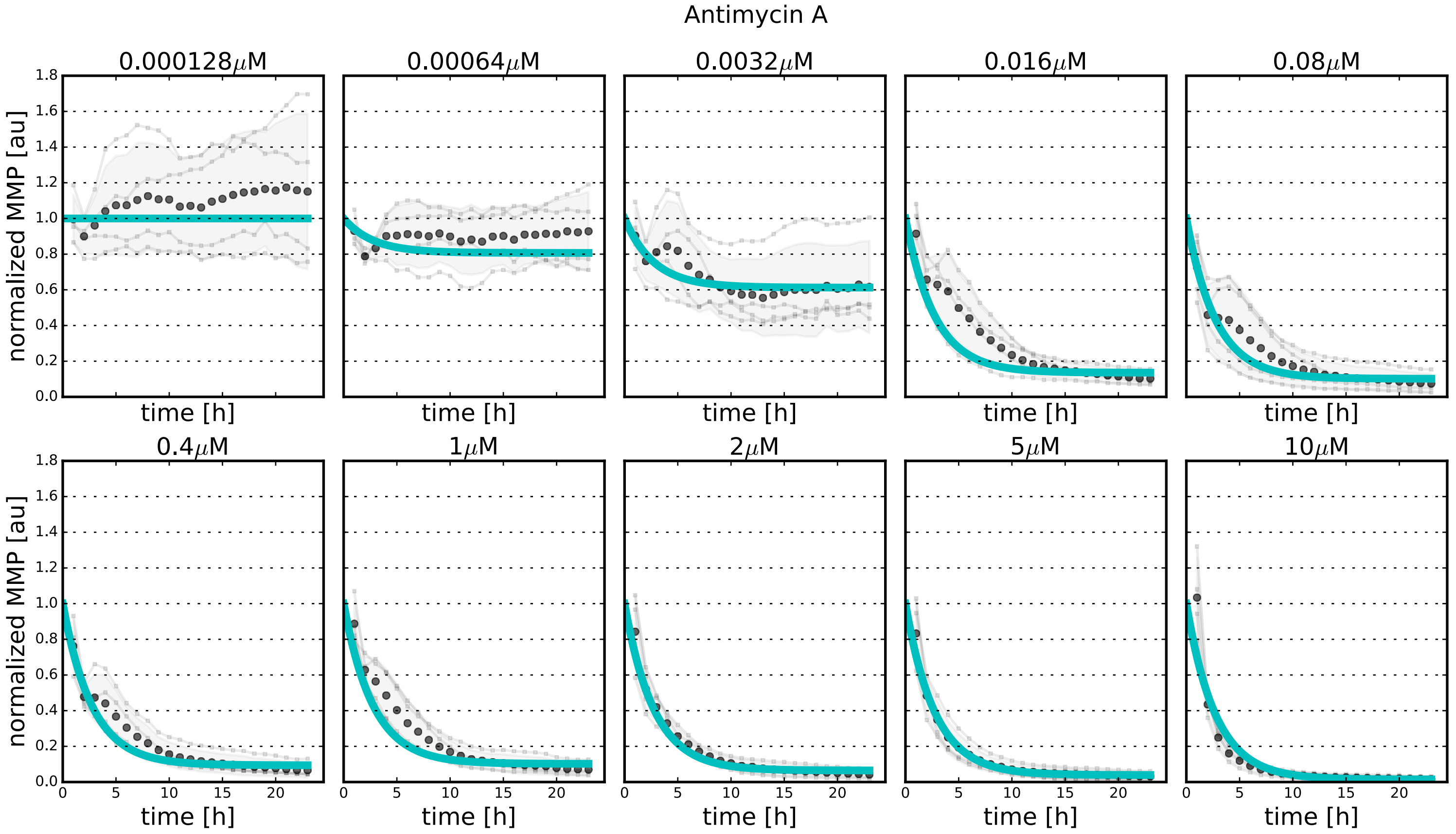

Supplementary Figure 2: Phenomological model fit of mitochondrial membrane potential dynamics

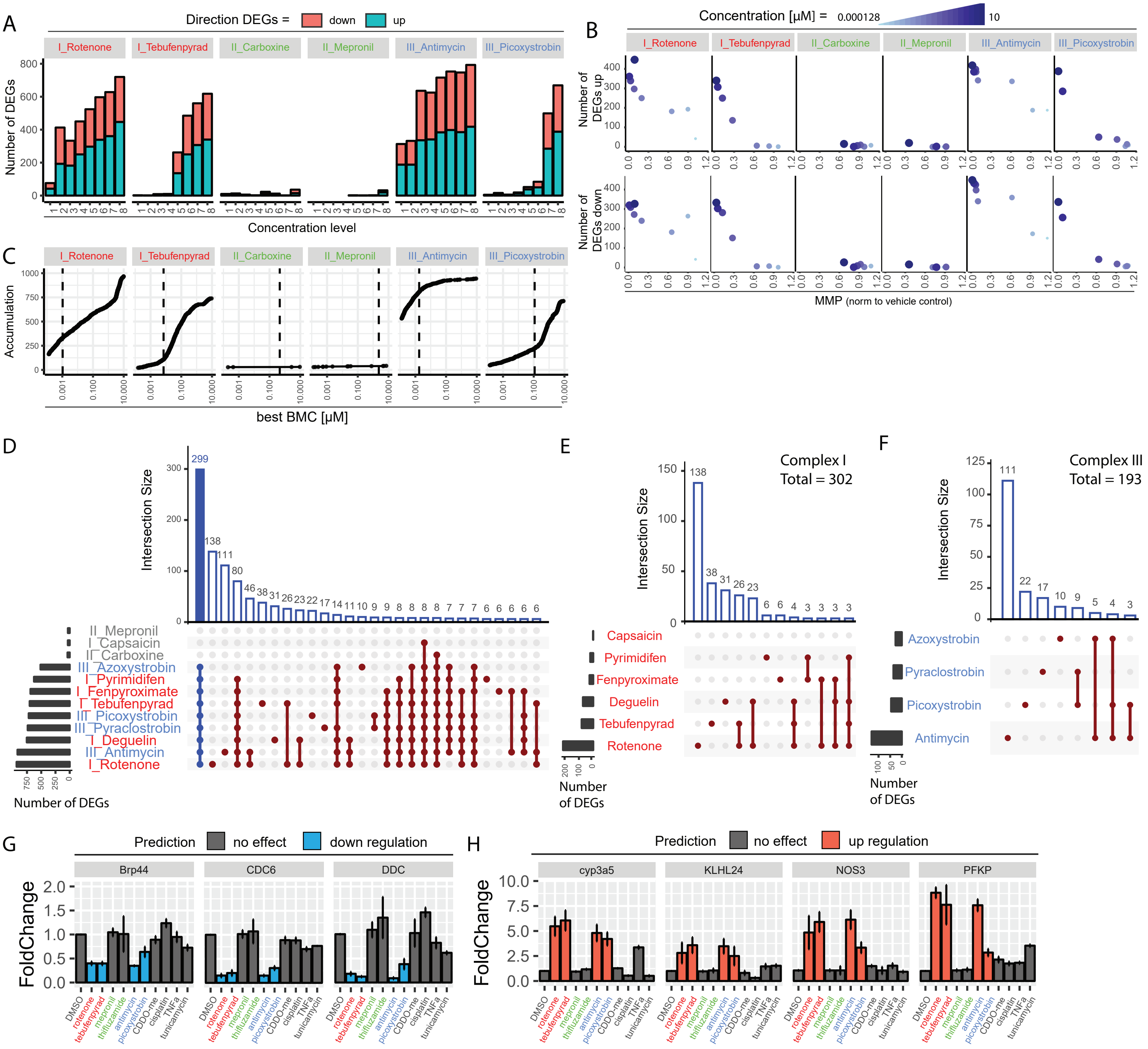

Supplementary figure 3: Predictive gene expression profiles for MRC inhibition

A

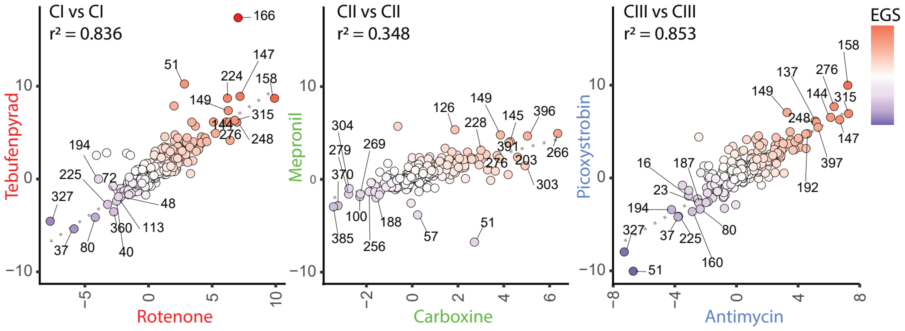

B

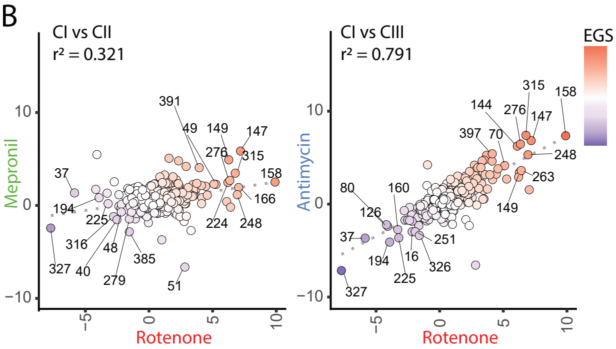

C

Module	Percentage covered	Pathways	Genes
144	5/11 (45.5%)	oxidative stress	**GCLM**, **TXNRD1**, **GPAT3**, **SRXN1**, DCX
147	6/11 (54.5%)	Protein metabolism, Insulin signaling	**NRP1**, **SPP1**, NAMPT, **IGF1**, RAB27A, NNMT
149	3/10 (30%)	Metabolism, mitochondrial trafficking	**IGFBP1**, **TOMM34**, **S100A16**
158	6/10 (60%)	MAPK signaling	**DUSP1**, **KLF6**, **CYR61**, **MYC**, **JUN**, TIPARP
248	4/7 (57.1%)	Signal transduction, metabolism	**AHR**, **NR3C1**, **CBLB**, **FZD5**
276	3/7 (42.9%)	Signaling (JNK, lipophilic components)	**LRP10**, **FNDC4**, DUSP22
315	1/6 (16.7%)	Vesicle transport	**CLTB**

D

ETC inhibitor	Abb.	Complex	MMP BMD [μM]	Selected conc [μM]	Highest non-toxic conc [μM]
Capsaicin	CAP	I	NA	10	10
Deguelin	DEG	I	0.04758*	0.016	10
Fenpyroximate	FPX	I	0.00067	0.0032	10
Pyrmidifen	PMD	I	0.00028	0.00064	10
Rotenone	ROT	I	0.00106	0.0032	2
Tebufenpyrad	TEB	I	0.00704	0.016	10
Carboxine	CAR	II	0.46302	2	10
Mepronil	MEP	II	2.33739*	10	10
Thifluzamide	THI	II	1.76686*	2	10
Antimycin A	AA	III	0.00177*	0.0032	10
Azoxystrobin	AZO	III	0.44700	2	10
Cyazofamid	CYA	III	4.82265	10	10
Picoxystrobin	PIC	III	0.11466*	0.4	10
Pyraclostrobin	PYR	III	0.20586	0.4	10

Supplementary Figure 4: Gene network activation by MRC inhibitors based on human hepatocyte WGCNA datasets

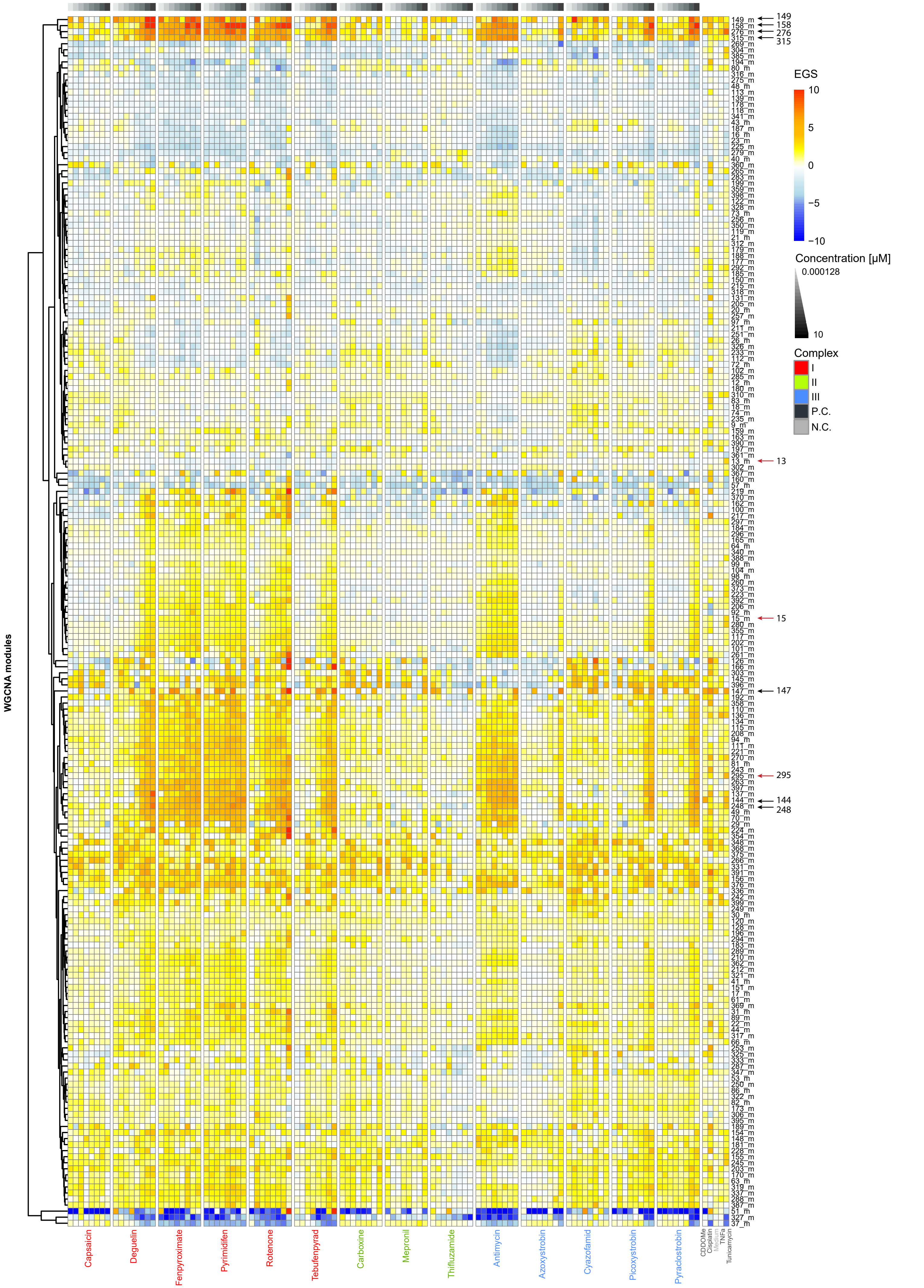

Supplementary Figure 5: Gene network activation by MRC inhibitors based on human hepatocyte WGCNA datasets

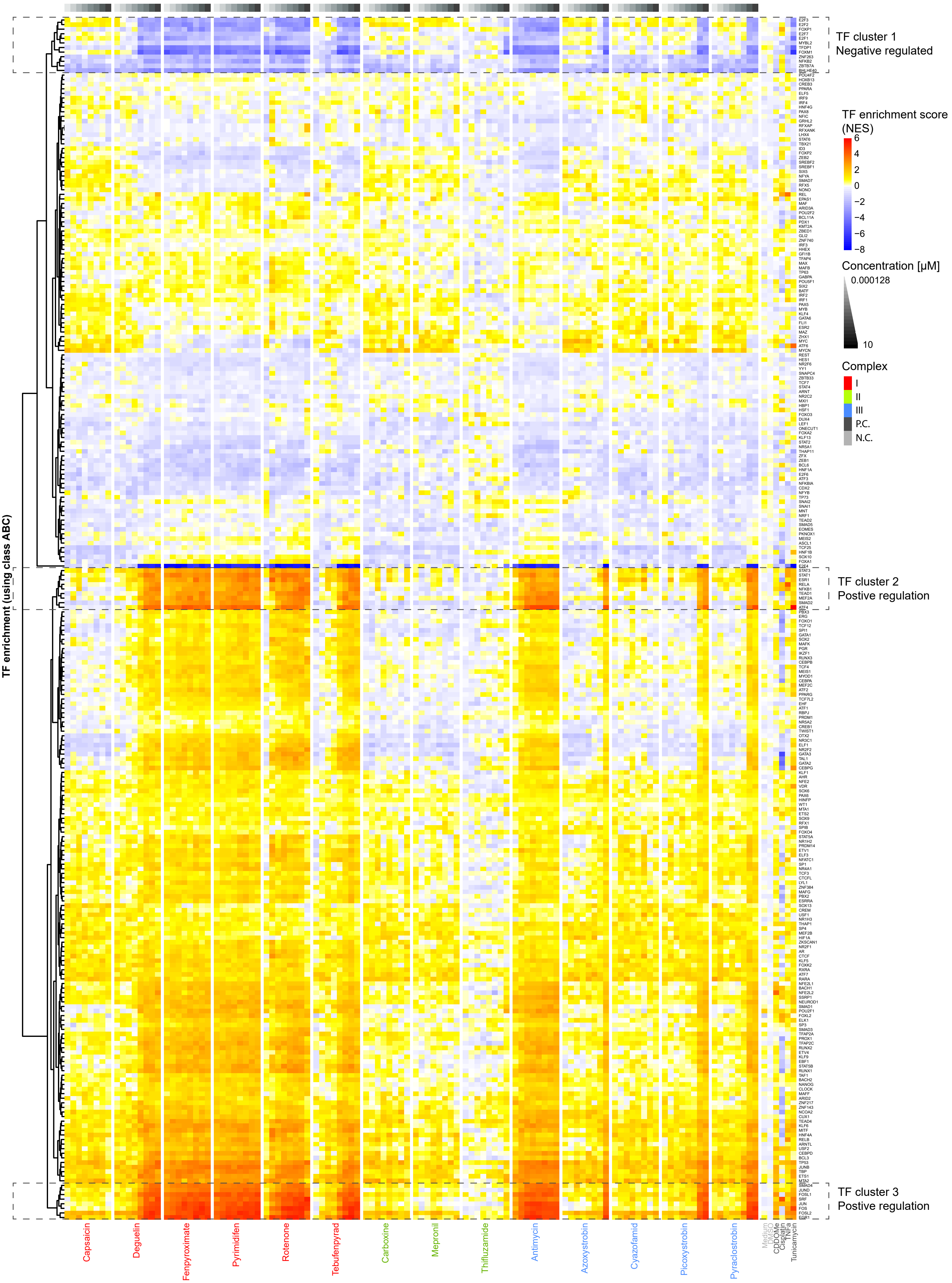

Supplementary Figure 6: Transcriptional reprogramming after treatment with agrochemical MRC inhibitors

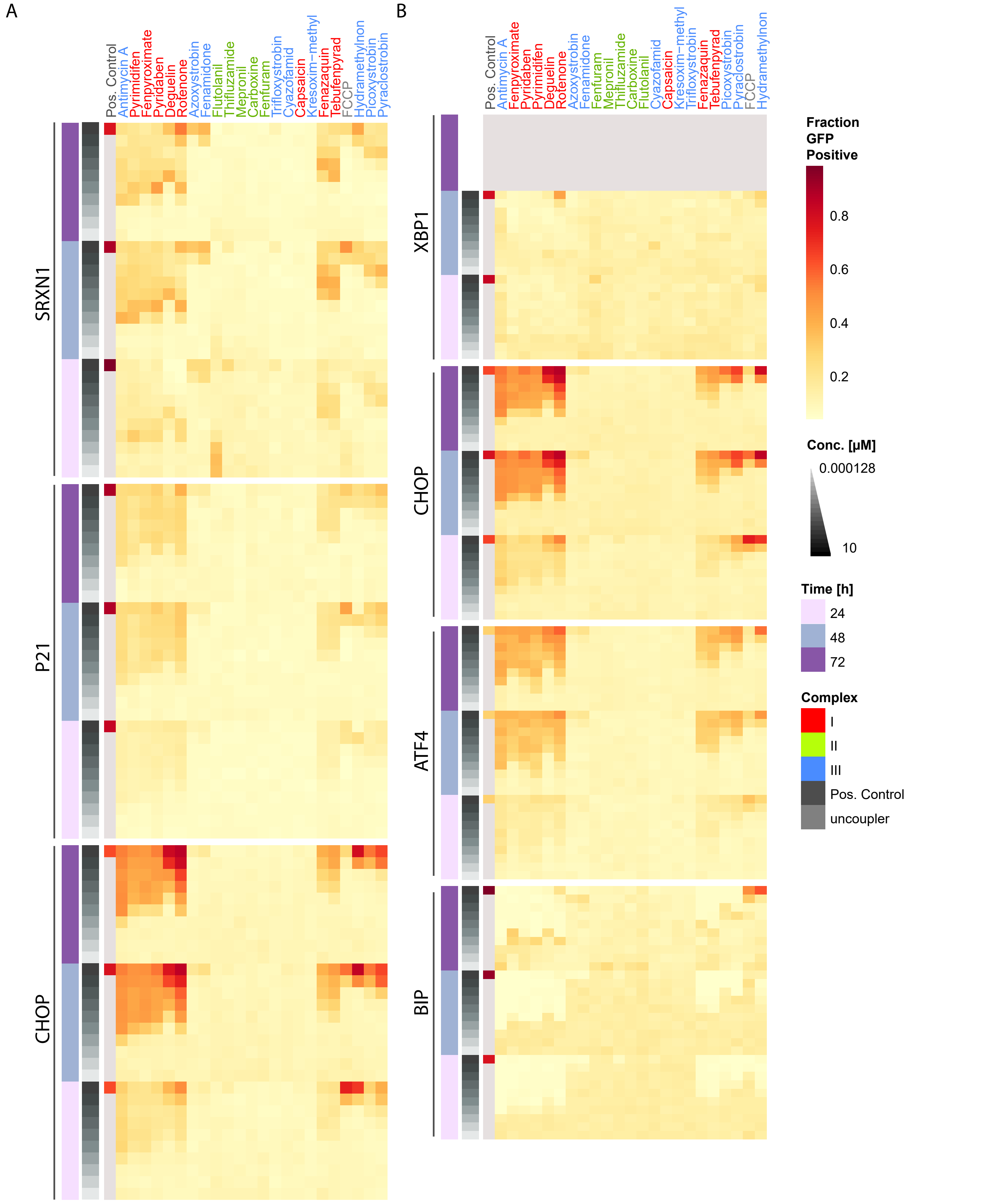

Supplemantary Figure 7: Effects of various agrochemical MRC inhibitors on cellular stress response reporter activation
