# Supplementary material for: Mapping the cellular response to electron transport chain inhibitors reveals selective signaling networks triggered by mitochondrial perturbation
Source: Arch Toxicol. 2021 Oct 13;96(1):259–85. doi: 10.1007/s00204-021-03160-7 (PMC8748354; doi:10.1007/s00204-021-03160-7)
Supplement: Supplementary file 2 — Supplementary file2 (DOCX 15 KB) [file 204_2021_3160_MOESM2_ESM.docx]

**Supplementary table 1: ATP biosensor, CFP and YFP constructs**

| Plasmid | Origin | Localization |
| --- | --- | --- |
| pcDNA-ateam1.03 | Gift Imamura | Cytosol |
| pcDNA-mitAT1.03 (pcDNA-CoxVIII2-AT1.03) | Gift Imamura | Mitochondria |
| pcDNA-Ateam 1.03 R122K-R126K | Gift Imamura | Cytosol |
| pECFP-C1 | Clonetech | Cytosol |
| pEYFP-C1 | Clonetech | Cytosol |

**Supplementary table 2: TempO-Seq Probe list**

Table depicting all probes in the S1500++ with information concerning the related gene name, EnsemblID, and if they were in the original S1500 [Mav 2018].

**Supplementary table 3: RT-qPCR primers**

| Gene symbol | Forward (F)/ | Sequence |
| --- | --- | --- |
|  | Reverse (R) |  |
| Brp44/MPC2 | F | CTACAGGGTTTATTTGGTCAAG |
|  | R | AATACGAAAAAGCTGAGAGG |
| CDC6 | F | ATGTAAATCACCTTCTGAGC |
|  | R | GTCATCCTGTTACCATCAAC |
| Cyp3a5 | F | CTCCTCTATCTATATGGGACC |
|  | R | ATACGTTCCCCACATTTTTC |
| DCAF6 | F | CACTTATCATATCCGAGCTG |
|  | R | TAAGTGTTGCCAAAAAGAG |
| DDC | F | GCTGCAGGAATCAAAAATTG |
|  | R | CAACCCTCTGGATAACTTTG |
| KLHL24 | F | AAAGTATATGTTGTCGGTGG |
|  | R | AGTATTATCATCAGGTCCTCC |
| NOS3 | F | CATCACCTATGACACCCTC |
|  | R | AGCCGCTCCTCTTAATG |
| PFPK | F | ATTTGTGTGCTGGGAATAAG |
|  | R | GGAATCCTGTGCTCAAAATC |
